# A clathrin-related protein FaRRP1/SCD2 integrates ABA trafficking and signaling to regulate strawberry fruit ripening

**DOI:** 10.1016/j.jbc.2023.105250

**Published:** 2023-09-13

**Authors:** Jiajing Li, Yuanyue Shen

**Affiliations:** College of Plant Science and Technology, Beijing University of Agriculture, Beijing, China

**Keywords:** abscisic acid (ABA), clathrin-mediated endocytosis, FaRRP1/SCD2, ABA receptor, ABAR, PYL2, signaling transduction, strawberry fruit ripening

## Abstract

Abscisic acid (ABA) is a critical regulator for nonclimacteric fruit ripening such as in the model plant of strawberry (*Fragaria* × *ananassa*). Although FaRRP1 is proposed to participate in clathrin-mediated endocytosis of ABA, its action molecular mechanisms in ABA signaling are not fully understood. Here, using our isolated FaRRP1 (ripening-regulation protein) and candidate ABA receptor FaPYL2 and FaABAR from strawberry fruit, a series of *silico* and molecular interaction analyses demonstrate that they all bind to ABA, and FaRRP1 binds both FaPYL2 and FaABAR; by contrast, the binding affinity of FaRRP1 to FaPYL2 is relatively higher. Interestingly, the binding of FaRRP1 to FaPYL2 and FaABAR affects the perception affinity to ABA. Furthermore, exogenous ABA application and *FaRRP1* transgenic analyses confirm that FaRRP1 participates in clathrin-mediated endocytosis and vesicle transport. Importantly, FaRRP1, FaPYL2, and FaABAR all trigger the initiation of strawberry fruit ripening at physiological and molecular levels. In conclusion, FaRRP1 not only binds to ABA but also affects the binding affinity of FaPYL2 and FaABAR to ABA, thus promoting strawberry fruit ripening. Our findings provide novel insights into the role of FaRRP1 in ABA trafficking and signaling, at least in strawberry, a model plant for nonclimacteric fruit ripening.

Fruit ripening is a significant and complex process tightly linked to fruit quality (1, 2, 3). Depending on the presence or absence of the peak in respiratory burst and ethylene release during ripening, fleshy fruits can be classified into climacteric and nonclimacteric types (3). Based on the model of climacteric tomato fruit, great progress has been made in understanding climacteric fruit ripening by ethylene perception and signaling transduction (4). However, by contrast, the nonclimacteric fruit ripening shows a more complicated process and is not fully understood (5, 6). Although in the past decade, increasing evidence uncovers a vital role of phytohormone abscisic acid (ABA) in nonclimacteric fruit ripening, especially establishing two core signaling pathways in strawberry fruit, as a model for nonclimacteric fruit ripening (2, 7, 8, 9, 10, 11, 12, 13), however, a substantial integration of the two ABA core signaling pathways is lacking.

It is known that an early classical report suggests an essential role of ABA in the ripening of nonclimacteric fruit based on the onset of grape berry ‘veraison’ (13), especially in the past decade, a line of breakthroughs have been made toward understanding the role of ABA in nonclimacteric fruits by a series of substantial molecular pieces of evidence in strawberry (2, 3, 7, 14, 15, 16). For example, the downregulation of the strawberry ABA biosynthetic key gene (*9-cis epoxycarotenoid dioxygenase*: *FaNCED1*) and putative ABA receptor gene (*magnesium chelatase H subunit*, *FaCHLH/ABAR*), both inhibit fruit ripening (10). An R2R3 MYB transcription factor10 (MYB10) is a downstream component of light and FaCHLH/ABAR in anthocyanin accumulation (17). Notably, the N6-methyladenosine modification enhances the mRNA stability of *NCED5* and *AREB1* (*ABA-responsive element-binding protein1*), finally promoting the translation efficiency of ABAR in strawberry fruit, demonstrating that the N6-methyladenosine methylation may positively regulate the ripening of the nonclimacteric strawberry fruit by ABA (2). Also, a strawberry leu-rich repeat receptor-like kinase, red-initial protein kinase 1 (FaRIPK1), is demonstrated to interact with FaABAR and positively regulates the fruit ripening, suggesting a synergistic action of FaRIPK1 with FaABAR (15). Also, a recent report finds that FvRIPK1 interacts with FvSnRK2.6 and phosphorylates each other (16), supporting a link between ABAR/CHLH and SnRK2.6/OST1 in guard cell signaling in response to ABA (18). Altogether, these reports support the proposed ABA-ABAR-RIPK1-SnRK2.6-ABI4 (ABA-insensitive 4) model in the control of strawberry fruit ripening (7, 8).

Notably, PYR1/PYL/RCAR family proteins (PYLs), as well-characterized ABA receptors, contain 14 PYL members in *Arabidopsis thaliana*, apart from PYL13 without binding to ABA; in contrast, all PYLs may inhibit the phosphatase activity of PP2CA (19). Also, the action of PYL2 and PYL10 rely on ABA to inhibit PP2CA (19, 20). In addition, PYLs have different oligomeric states, including dimeric PYR1/PYL1-2, monomeric PYL4-12, and monomer-dimer exchanging PYL3; interestingly, the dimeric receptors appear intrinsically less sensitive to ABA than the monomeric receptors, for example, the monomeric mutant PYL2-I88K increases a 7-fold higher affinity to ABA than its natural receptor (20, 21). In short, the monomers have higher ABA binding affinity, while the dimers have relatively lower ABA binding. A recent study reports that the binding of ABA destabilizes the PYL2 complex and further stabilizes the association of PYL2-HAB1 (hypersensitive TO ABA1), thus promoting PYL2 dissociation (22). Indeed, ABA-bound PYL2 serves as a competitive inhibitor of PP2Cs in the ternary ABA-PYL2-HAB1 complex; especially among 14 members of the PYR/PYL family, the SGLPA and HRL amino acid sequences are conserved (23).

At the structural level, PYLs contain a pocket surrounded by four highly-conserved surface loops (CL1-4), among which CL2 may close the pocket to create a surface for ABI1 (ABA insensitive 1) recognition in response to ABA binding; as a result, ABA-bound PYLs inhibit PP2Cs by blocking the entry of substrate SnRK2s (24, 25, 26, 27, 28). For example, ABA directly binds PYL2 by the carboxylate of ABA and Lys-64 of PYL2 (also including Val-87, Leu-91, Pro-92, and Ala-93), while the hydroxyl group of ABA only recognized PYL2 through a water-mediated hydrogen bond. Also, the residue Ser-89 of PYL2 (corresponding to Ser-112 in PYL1) plays a crucial part in interacting with ABI1 (24, 25). Similarly, the residues of PYL1 in the recognition of ABA are involved in several core amino acids with dissociation constant (Kd) values of 52 to 340 μM (26). Notably, the binding affinity of ABA with PYL9 (RCAR1) may increase ten-fold in the presence of phosphatase ABI2 (27). In summary, the structural mechanisms of ABA-PYL recognition include the following: (1) the carboxyl of ABA can form a salt bridge by the key amine group of lysine (PYR1 K59, PYL1 K86, PYL2 K64, PYL3 K79, PYL9 K63, and PYL10 K56) and a water-mediated hydrogen bond network; (2) one ABA binds to the receptor, the residue on the “gate” loop (PYR1 P88, PYL1 P115, PYL2 P92, and PYL3 P112) moves toward the pocket to close the “gate” loop; (3) the residue (PYR1 S85, PYL1 S112, PYL2 S89, PYL3 S109) coordinately flips outward the cavity, and their imidazole group of the residue on the “latch” loop (PYR1 H115, PYL1 H142, PYL2 H119, PYL3 H139) orientates inward the cavity to contact ABA (28). These advances help us to better understand the action mechanism of PYLs.

Notably, it has been previously reported that stomatal cytokinesis defective 2 (SCD2) functions in Arabidopsis cytokinesis and cell expansion through the clathrin-mediated plasma membrane (PM) endocytosis and clathrin-coated vesicles (29). The homolog of SCD2, a strawberry ripening-regulated protein 1 (FaRRP1, GenBank: JQ619656.1), is potentially involved in strawberry fruit ripening (30, 31). Also, a model for SCD2/RRP1-mediated ABA signaling is proposed: when ABA contents are low or absent, RRP1 may bind and activate ABI1 activity and, as a result, promoting the inhibition of SnRK2 activity and ABA response; when ABA contents are high, ABA binds RRP1, promoting the RRP1-mediated PYR1 and ABI1 to move to the PM for formatting a three-protein complex, which is potentially associated to the SCD2/RRP1-mediated endocytosis of ABA and ABA signaling (30). However, the RRP1-mediated ABA signaling pathway in fruit ripening remains yet unknown.

Given strawberry *FaPYR/PYL* and *FaPP2C* gene families, both have at least nine members, respectively, among which *FaPYL2/4/8/9/11/12* and *FaABI1/FaPP2C16/51/16L2/16L1/37* showed relatively higher expression during ripening, and FaPYL2 and FaABI1 interaction may play a main role in the ripening (11). Thus, in the present study, to explore the relationships of FaRRP1 with FaPYL2, FaABAR, and ABA, we first measured the affinity and enthalpy in the binding of ABA to FaRRP1, FaPYL2, and FaABAR using isothermal titration calorimetric (ITC) assay, respectively; second, investigate the interaction of FaRRP1 separately with FaPYL2 and FaABAR in *silico* and further confirmed by a series of interaction analyses, including yeast two-hybrid (Y2H), pull-down, and bimolecular fluorescence complementation (BiFC), demonstrating that FaRRP1 not only separately interacts with FaPYL2 and FaABAR but also significantly affect the binding of FaPYL2 and FaABAR to ABA, respectively. Also, we observed that these genes initiated strawberry fruit ripening using the *Agrobacterium*-mediated fruit transformation method, and finally, FaRRP1-mediated cellular ABA uptake by clathrin-mediated endocytosis was investigated. In conclusion, the FaRRP1-mediated integration of two ABA signaling pathways may contribute to the fine-tuning regulation of strawberry fruit ripening by ABA trafficking and signaling, providing not only now insights into ABA in the regulation of nonclimacteric fruit ripening but also a potential strategy for fruit quality improvement next, at least in strawberry.

## Results

### Binding kinetics among FaRRP1, FaPYL2, and FaABAR to ABA

**Figure 1:**
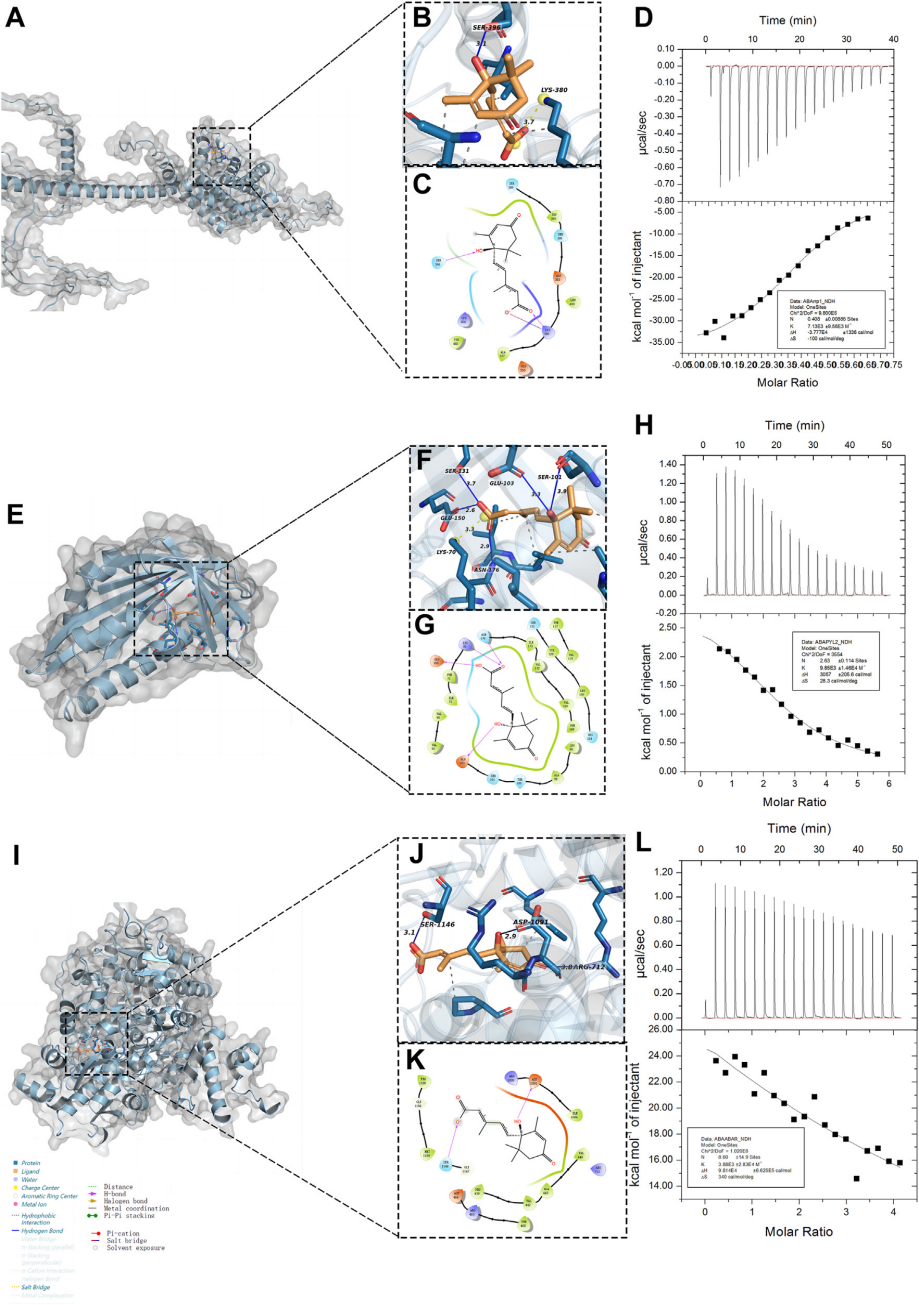
**Investigation of the bound affinity of FaRRP1, FaPYL2, and FaABAR to ABA using *silico* analysis and isothermal titration calorimetry.***A*, *E* and *I*, predict the 3D models of FaRRP1, FaPYL2, and FaABAR through AlphaFold2 v2.3. *B*, *F* and *J*, the combination pockets of FaRRP1, FaPYL2, and FaABAR with ABA were predicted by Schrödinger and mapped by the pyMOL module in a 3D structure. *C*, *G* and *K*, the combination pockets of FaRRP1, FaPYL2, and FaABAR with ABA were predicted by Schrödinger and mapped by the ligand interaction module in a 2D structure. *D*, 0.4 mM ABA titrate 20 μM FaRRP1 protein solution. *H*, 0.4 mM ABA titrate 20 μM FaPYL2 protein solution. *L*, 0.4 mM ABA titrate 20 μM FaABAR protein solution. Data are shown as the mean ± standard error (n = 3). ABA, abscisic acid.

**Supplemental Fig. S1:**
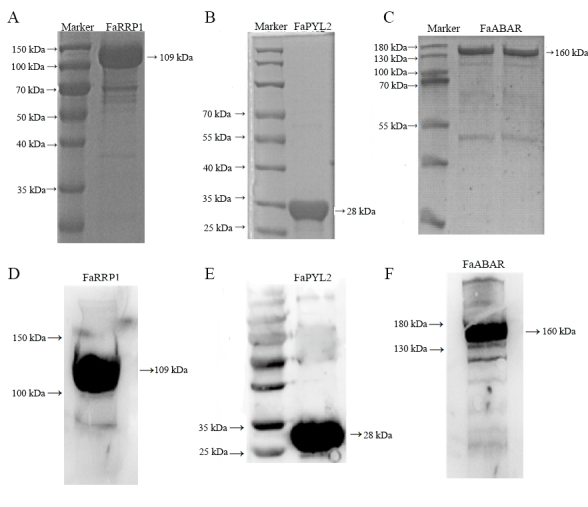
**SDS-PAGE and Western blot assays showing the determination of protein concentration and purification.***A–C*, FaRRP1-His/MBP, FaPYL2-His, and FaABAR-His recombinant fusion proteins were purified using SDS-PAGE analysis. *D* and *E*, Western blot was used to identify the recombinant fusion proteins of FaRRP1-His/MBP, FaPYL2-His, and FaABAR-His. Marker: 10 ∼ 180 kDa.

**Supplemental Fig. S2:**
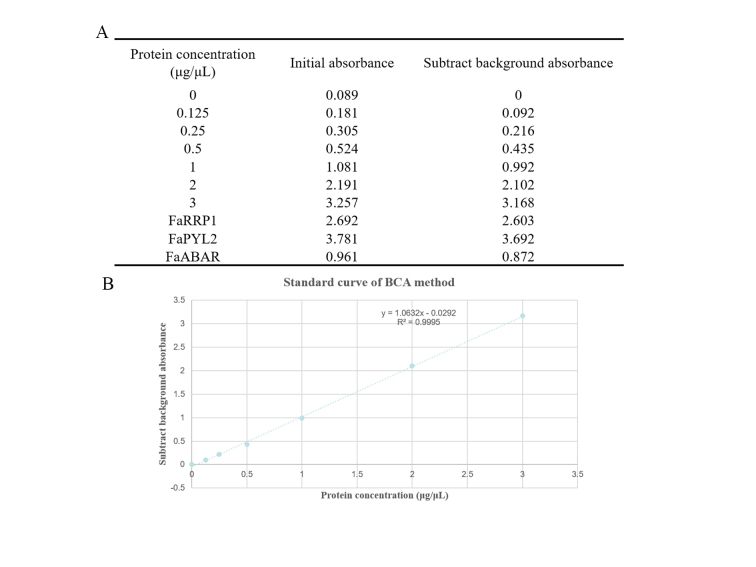
**Standard curve for determination of protein concentration by BCA.***A*, different BSA Standard samples concentration absorbance at 562 nm. *B*, standard curve for protein quantification.

**Supplemental Fig. S3:**
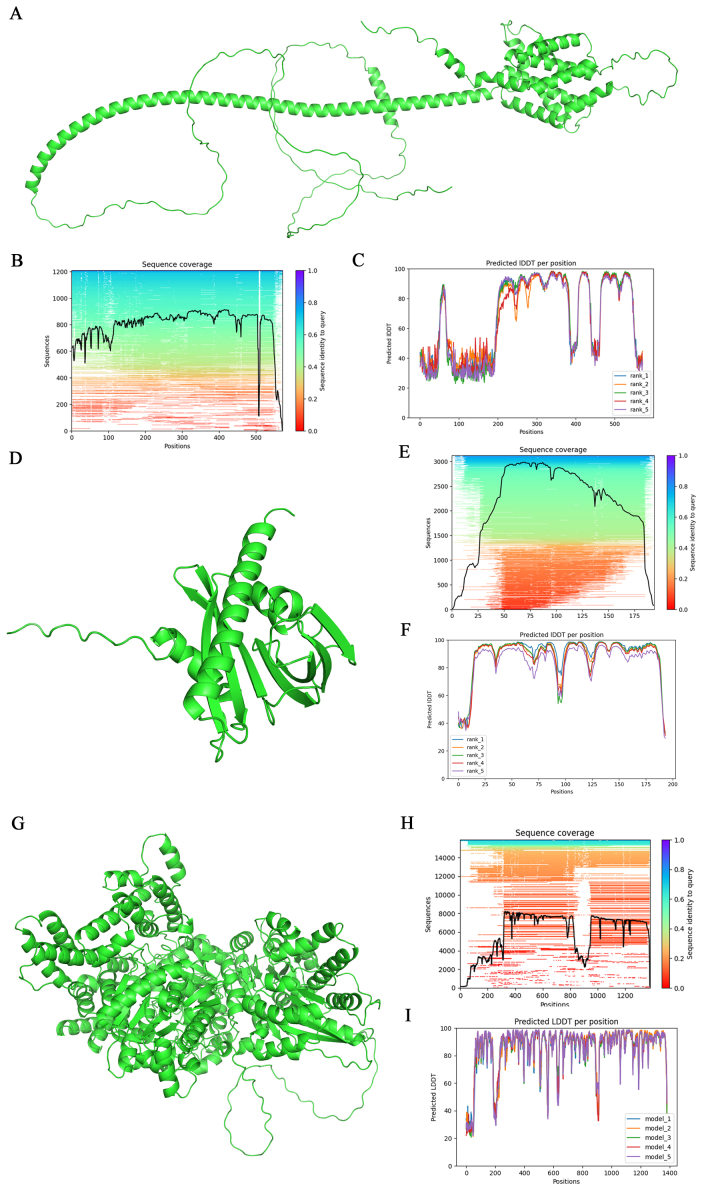
**Structural of FaRRP1, FaPYL2, and FaABAR predicted by AlphaFold.***A*, *D* and *G*, global view of the structure of FaRRP1, FaPYL2, and FaABAR. *B*, *E* and *H*, sequence coverage of FaRRP1, FaPYL2, and FaABAR proteins was compared with the database. *C*, *F* and *I*, pLDDT values of amino acid residues in FaRRP1, FaPYL2, and FaABAR proteins.

**Supplemental Fig. S4:**
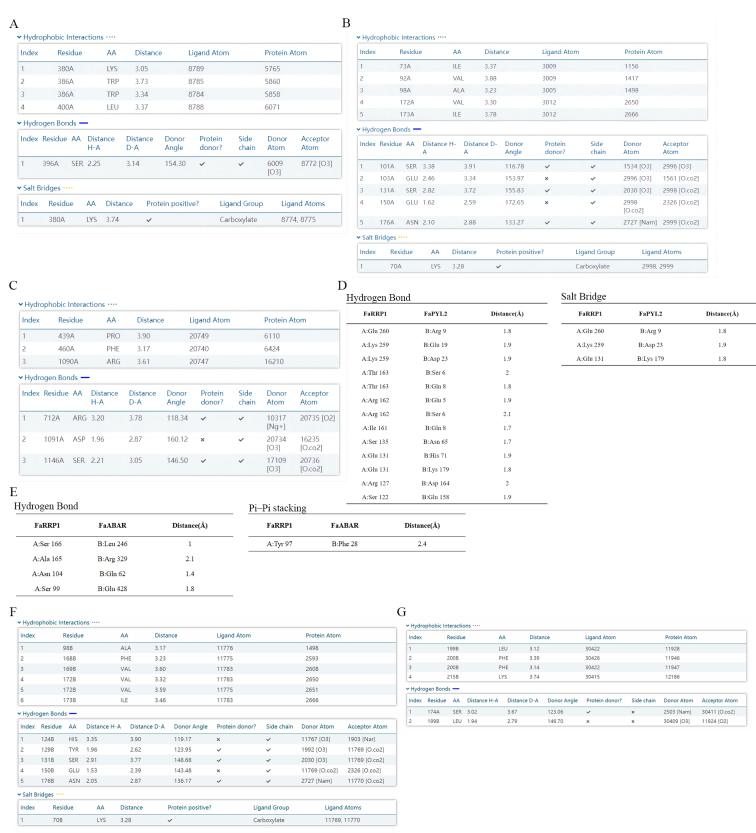
**Analyze the interactions of various molecules.***A*, FaRRP1 binding sites with ABA and various interaction forces. *B*, FaPYL2 binding sites with ABA and various interaction forces. *C*, FaABAR binding sites with ABA and various interaction forces. *D*, interaction analysis of FaRRP1 and FaPYL2. *E*, interaction analysis of FaRRP1 and FaABAR. *F*, interaction of FaRRP1-FaPYL2 protein complex with ABA formation. *G*, interaction of FaRRP1-FaABAR protein complex with ABA formation.

To investigate the binding kinetics of ABA to the three proteins, first, the purification of FaRRP1-His/MBP, FaPYL2-His, and FaABAR-His recombinant fusion proteins was carried out using both SDS-PAGE (Fig. S1, *A–C*) and Western blot (Fig. S1, *D–F*) analyses. Subsequently, the protein concentration was measured using the bicinchoninic acid (BCA) method (32), and the standard curve was done and showed in the supplementary material (Fig. S2, *A* and *B*). The purified protein concentrations of FaRRP1, FaPYL2, and FaABAR were 2.738, 3.89, and 0.989 μg/μl, respectively, then they all were adjusted to 20 μM (15) for subsequent analyses. Second, according to the previous report on AlphaFold2 protein structure prediction (32), we predict the 3D models of FaRRP1, FaPYL2, and FaABAR through AlphaFold2 v2.3 and analyze the predicted results (Fig. S3, *A–I*), respectively. We select the model with the highest confidence (model/rank 1) for assessments (Fig. S3, *A*, *D* and *G*). Subsequently, we use semiflexible docking of the binding pockets of FaRRP1, FaPYL2, and FaABAR for ABA matching prediction by Schrödinger and mapped by pyMOL and ligand interaction module (Fig. 1, *A*–*C*, *E–G*, *I–K*). We found that the active pocket of FaRRP1 with ABA was mainly located in amino acid residues from Ala-377 to Ser-389. In addition, Ser-396 could also interact with ABA (Fig. 1, *A*–*C*). Similarly, the active pocket of FaPYL2 with ABA was located in Lys-70 to Ile-73 and Glu-103 to Asn-176, among which Glu-150 could interact with ABA (Fig. 1, *E*–*G*). The active pocket of FaABAR with ABA was located in Pro-439 to Val-446, Arg-1090 to Ile-1094, Ser-1146 to Gly-1147, and Pro-1185 to Met-1189 (Fig. 1, *I–K*). Further observation found that ABA could form hydrogen bonds with FaRRP1’s Ser-396 and Lys-380; FaPYL2’s Ser-131, Glu-150, Glu-103, Ser-101 and Asn-176; and FaABAR’s Ser-1146, Asp-1091 and Arg-712 (blue solid line; Fig. 1, *B*, *F* and *J*), respectively. Also, ABA could form an ionic interaction separately with Lys-380 of FaRRP1 and Lys-70 of FaPYL2 (yellow dashed line; Fig. 1, *B*, *F* and *J*), which also linked to multiple hydrophobic interactions (gray dashed line; Fig. 1, *B*, *F* and *J*). Altogether, these data suggest that FaRRP1, FaPYL2, and FaABAR may interact with ABA through the pocket-residue analyses (Fig. S4, *A–C*).

In addition, we further observed the interrelationships of FaRRP1, FaPYL2, and FaABAR with ABA in the two-dimensional diagram: (1) Ser-396 of FaRRP1 formed hydrogen bonds with the hydroxyl group (-OH) of ABA, and Lys-380 formed ionic interaction and hydrogen bonds with the hydroxyl group (-OH) and a carbonyl group (C=O) on the carboxyl group (-COOH) of ABA (Fig. 1, *B* and *C*), respectively; (2) Glu-103 and Glu-150 of FaPYL2 formed hydrogen bonds with the -OH of ABA, and its Lys-70 and Asn-176 formed hydrogen bonds with the -COOH of ABA (Fig. 1, *F* and *G*); (3) Asp-1091 of FaABAR formed hydrogen bonds with the -OH of ABA, and the Ser-1146 forms hydrogen bonds with the -OH on the -COOH of ABA (Fig. 1, *J* and *K*). These results suggest a central role of Ser, Asp, Glu, and Lys in ABA binding.

**Supplemental Fig. S5:**
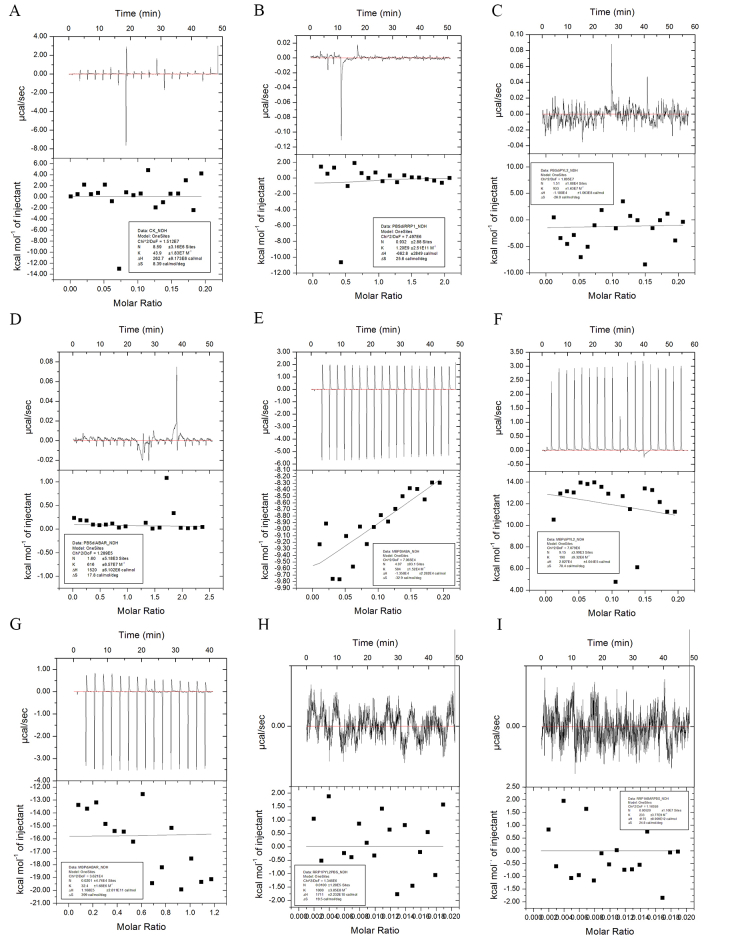
**Using isothermal titer calorimetry to determine protein with PBS buffer or the combination of ABA and MBP label protein affinity.***A*, 0.4 mM ABA titrate PBS buffer solution. *B–D*, PBS buffer titrate 20 μM FaRRP1, FaPYL2, and FaABAR protein solution. *E*, 0.4 mM ABA titrate 20 μM pMALC5X-His/MBP protein solution. *F*, 0.4 mM FaPYL2 protein solution titrate 20 μM pMALC5X-His/MBP protein solution. *G*, 0.4 mM pMALC5X-His/MBP protein solution titrate 20 μM FaABAR protein solution. *H*, PBS buffer titrate 20 μM FaRRP1-FaPYL2 protein complexes solution. *I*, PBS buffer titrates 20 μM FaRRP1-FaABAR protein complexes solution.

Given an essential role of the hydroxyl (-OH) group beneficial to molecule function (33), we proved the binding of FaRRP1, FaPYL2, and FaABAR to ABA by the biochemical experiment of ITC analyses using a 1-fold protein peptide and 20-fold S- (+)-ABA (Fig. 1, *D*, *H* and *L*). The results showed that the binding of FaRRP1 to ABA was an exothermic reaction (Fig. 1*D*), while FaPYL2 (Fig. 1*H*) and FaABAR (Fig. 1*L*) were an endothermic reaction in ABA binding, showing that the binding of FaRRP1 and FaPYL2 to ABA followed a saturation curve with a Kd of 140.2 μM and 101.5 μM, respectively; whereas, the binding of FaABAR to ABA could not reach saturation curve with a Kd of 257.7 μM. Thus, FaABAR binds to ABA with low affinity in our experimental situations. In addition, we performed a series of controlled experiments to determine the possible effect of other factors on ABA, finding that PBS Buffer (20 mM Na_2_HPO_4_, 20 mM NaH_2_PO_4_, 300 mM NaCl, 20 mM MgCl_2_ pH 7.4) and pMALC5X- His/MBP did not affect ABA-protein binding (Fig. S5, *A–E*). These data demonstrated that FaPYL2, FaRRP1, and FaABAR, more or less, all bind to ABA.

### FaRRP1 interacts with FaPYL2 and FaABAR, and the binding affinity of FaRRP1 to FaPYL2 was higher

**Figure 2:**
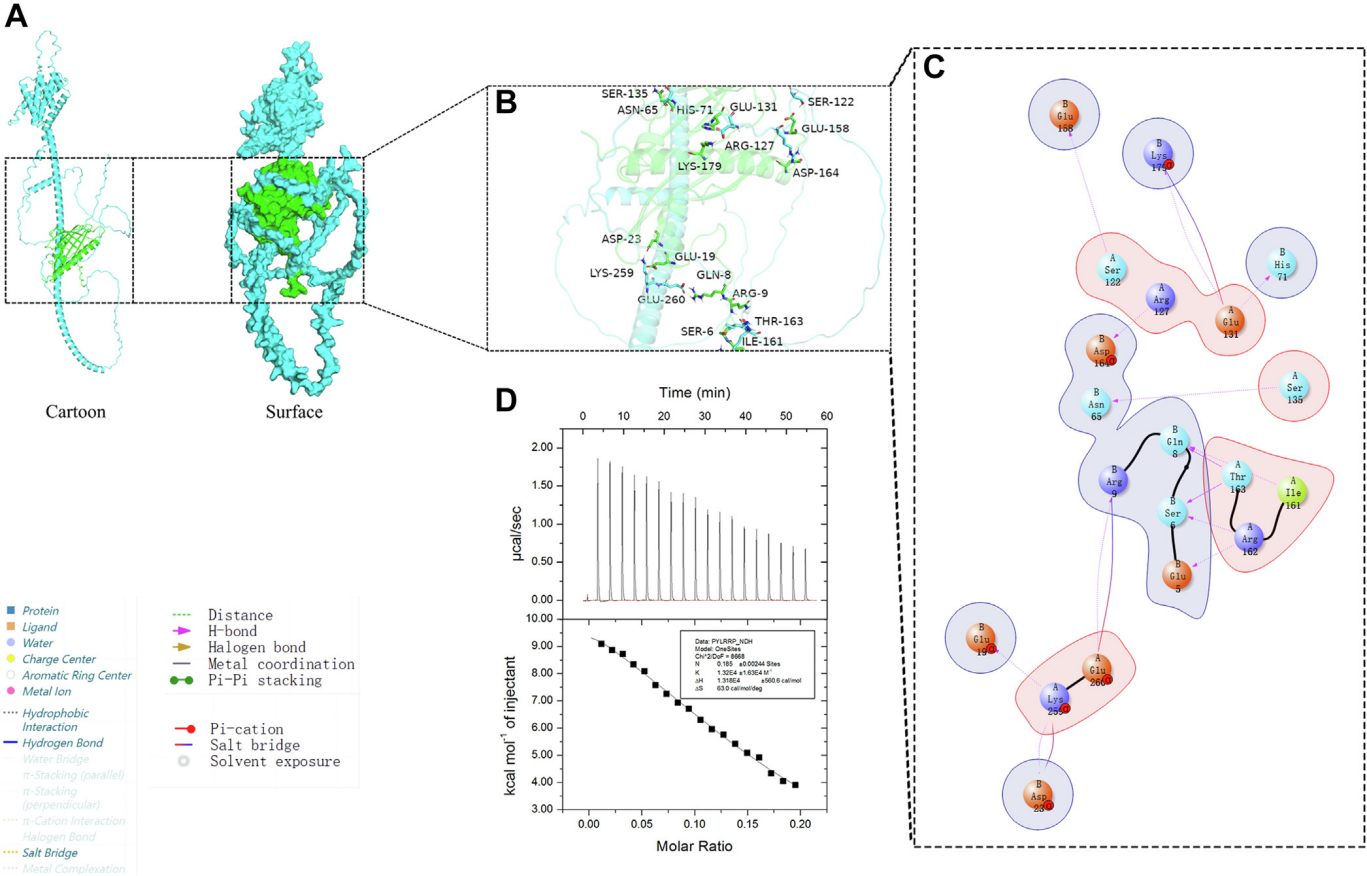
**The interaction sites between FaRRP1 and FaPYL2 were predicted by Schrödinger and pyMOL software analysis.***A*, cartoon and surface drawings of molecular docking of FaRRP1-FaPYL2. *B* and *C*, three-dimensional structure diagram and two-dimensional structure diagram of molecular docking of FaRRP1-FaPYL2. *A*: FaRRP1; *B*: FaPYL2; α: Alpha chain. *D*, measurement of the binding affinity between purified FaPYL2 and the purified FaRRP1 using isothermal titration calorimetry. Data are shown as the mean ± standard error (n = 3).

**Figure 3:**
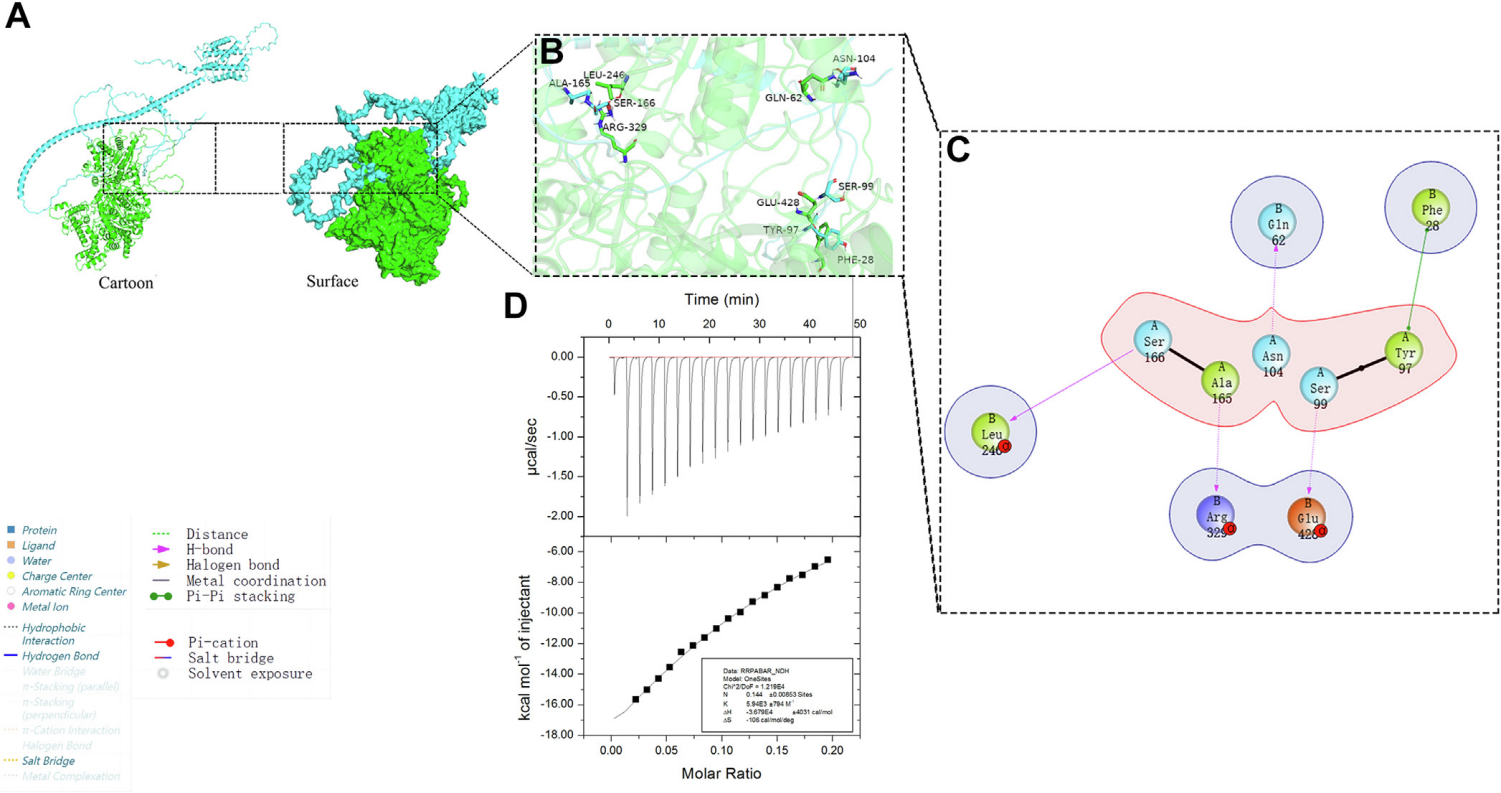
**The interaction sites between FaRRP1 and FaABAR were predicted by Schrödinger and pyMOL software analysis.***A*, cartoon and surface drawings of molecular docking of FaRRP1-FaABAR. *B* and *C*, three-dimensional structure diagram and two-dimensional structure diagram of molecular docking of FaRRP1-FaABAR. *A*: FaRRP1; *B*: FaABAR; α: Alpha chain. *D*, measurement of the binding affinity between purified FaRRP1 and the purified FaABAR using isothermal titration calorimetry. Data are shown as the mean ± standard error (n = 3).

Although FaRRP1, FaPYL2, and FaABAR bind to ABA, the mechanism of their interactions remains unclear. Based on the previously obtained protein data bank files, the Protein Preparation Wizard module of Schrödinger software (https://www.schrodinger.com/) was used for protein structure analysis and Protein-Protein molecular docking (Piper). The first-ranked conformation was selected for data analysis (Figs. 2 and 3*A*) by pyMOL (Figs. 2 and 3, *B* and *C*). The results showed that FaRRP1 had a line of hydrogen bonds and ionic interactions separately with FaPYL2 and FaABAR in three-dimensional and two dimensions diagrams: (1) the hydrogen bond formation in FaRRP1 including Glu-260, Lys-259, Thr-163, Arg-162, Ile-161, Ser-135, Glu-131, Arg-127, Ser-122 sites; FaPYL2 including Arg-9, Glu-19, Asp-23, Ser-6, Gln-8, Glu-5, Ser-6, Asn-65, HIS-71, Lys-179, Asp-164 and Glu-158 sites (Fig. 2, *B* and *C*); (2) the ionic interaction formation in FaPYL2 including Glu-260, Lys-259, Glu-131 of FaRRP1 and Arg-9, Asp-23, Lys-179 (Fig. 2, *B* and *C*); and (3) amino acid residue analysis including the interaction between FaRRP1 and FaPYL2 (Fig. S4*D*). Similarly, as shown in Figure 3, there was hydrogen bond formation in FaRRP1, including Ser-166, Ala-165, Asn-104, and Ser-99 sites; in FaABAR, including Leu-246, Arg-329, Gln-62, and Glu-428 sites (Fig. 3, *B* and *C*), especially finding a Pi-Pi stacking between Thr-97 of FaRRP1 and Phe-28 of FaABAR (Fig. 3, *B* and *C*). The amino acid residue analysis of the interaction between FaRRP1 and FaABAR (Fig. S4*E*) suggests that FaRRP1 may interact with FaPYL2 and FaABAR.

Subsequently, to determine whether the FaRRP1 protein really bind to FaPYL2 and FaABAR, we investigate the binding strength of FaRRP1 with FaPYL2 and FaABAR by ITC using the purified proteins. The results showed that the binding of FaRRP1 to FaPYL2 followed an endothermic reaction with a Kd of 75.6 μM (Fig. 2*D*). In comparison, the binding of FaRRP1 to FaABAR fell into an exothermic reaction with a Kd of 168.3 μM (Fig. 3*D*), and the pMAL-C5X-His/MBP protein could not affect the binding of FaRRP1 to FaPYL2 and FaABAR (Fig. S5, *F* and *G*). These data preliminarily demonstrate that FaRRP1 can interact with both FaPYL2 and FaABAR.

**Figure 4:**
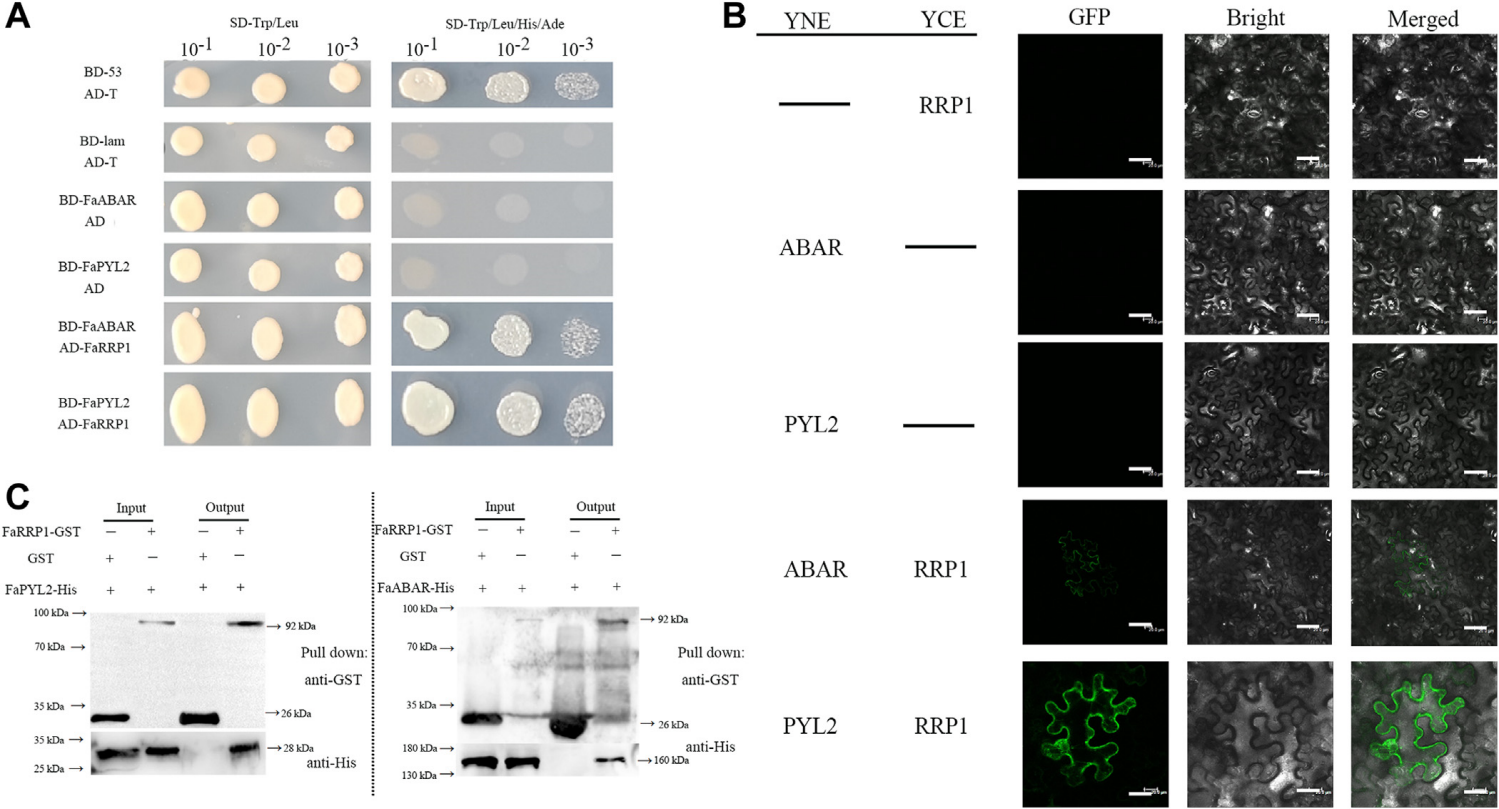
**The interaction and binding affinity of FaRRP1 with FaPYL2 and FaABAR.***A*, FaRRP1 interacts with FaPYL2 and FaABAR in the yeast two-hybrid (Y2H) assay. SD-Trp and Leu, synthetic dropout medium without Leu and Trp; SD−Leu, Trp, His, and Ade, synthetic dropout medium without Leu, Trp, His, and Ade; positive control, pGBKT7-53 (BD-53) + pGADT7-T (AD-T); negative control, pGBKT7-lam (BD-lam) + pGADT7-T (AD-T); self-activation, pGBKT7-FaABAR (BD-FaABAR) + pGADT7 (AD), pGBKT7-FaPYL2 (BD-PYL2) + pGADT7 (AD); experimental group, pGBKT7-FaABAR (BD-FaABAR) + pGADT7-FaRRP1 (AD-FaRRP1), pGBKT7-FaPYL2 (BD-FaPYL2) + pGADT7-FaRRP1 (AD-FaRRP1); 10-1, dilute 10×; 10-2, dilute 100×; 10-3, dilute 1000×. The experiment was performed with three replicates. *B*, the bimolecular fluorescence complementation (BiFC) assay indicates that FaRRP1 interacts with FaPYL2 and FaABAR; negative control, pSPYNE + pSPYCE-RRP1, pSPYNE-ABAR + pSPYCE, pSPYNE-PYL2 + pSPYCE. The experiment was performed with three replicates. Bars represent 20 μm. *C*, FaRRP1 interacted with FaPYL2 and FaABAR in GST pull-down assays. The ‘+’ and ‘−’ indicate the indicated protein's presence and absence, respectively. The experiment was performed with three replicates. GST, glutathione-S-transferase.

To further confirm the protein interaction above, we first performed Y2H analysis by cotransformed yeast cells with the AD-FaPRP1 vector and the BD-FaPYL2 or BD-FaABAR vector. The results showed that the transformed yeast cells grew on medium lacking Leu, Trp, His, and Ade, proving the interaction of FaRRP1 with both FaPYL2 and FaABAR (Fig. 4*A*). Second, we next performed a glutathione-S-transferase (GST) pull-down assay to further verify this interaction *in vitro* by the expressed and purified FaPYL2-His and FaABAR-His proteins, which were added to GST-FaRRP1 or GST protein and incubated for Western blot assay. GST pull-down assay showed that FaPYL2-His and FaABAR-His pulled down FaRRP1-GST but not by GST, reflecting an *in vitro* interaction between FaRRP1 with FaPYL2 and FaABAR (Fig. 4*C*). Third, we also performed BiFC test to confirm further the interaction *in vivo* by cotransforming *Nicotiana benthamiana* leaves of epidermal cells using the FaRRP1-_C_GFP plasmid and FaPYL2-_N_GFP or FaABAR-_N_GFP. The strong GFP signals detected the co-expressed FaRRP1-_C_GFP and FaPYL2-_N_GFP or FaABAR-_N_GFP in the epidermal cells, confirming their interactions *in vivo* (Fig. 4*B*).

Altogether, we provide a series of *in vitro* and *vivo* data to demonstrate that FaRRP1 interacts with FaPYL2 and FaABAR, and the binding affinity of FaRRP1 to FaPYL2 is higher than that of FaABAR, suggesting a central role of FaPYL2 in ABA perception.

### FaRRP1 affects the binding affinity of FaPYL2 and FaABAR to ABA

**Figure 5:**
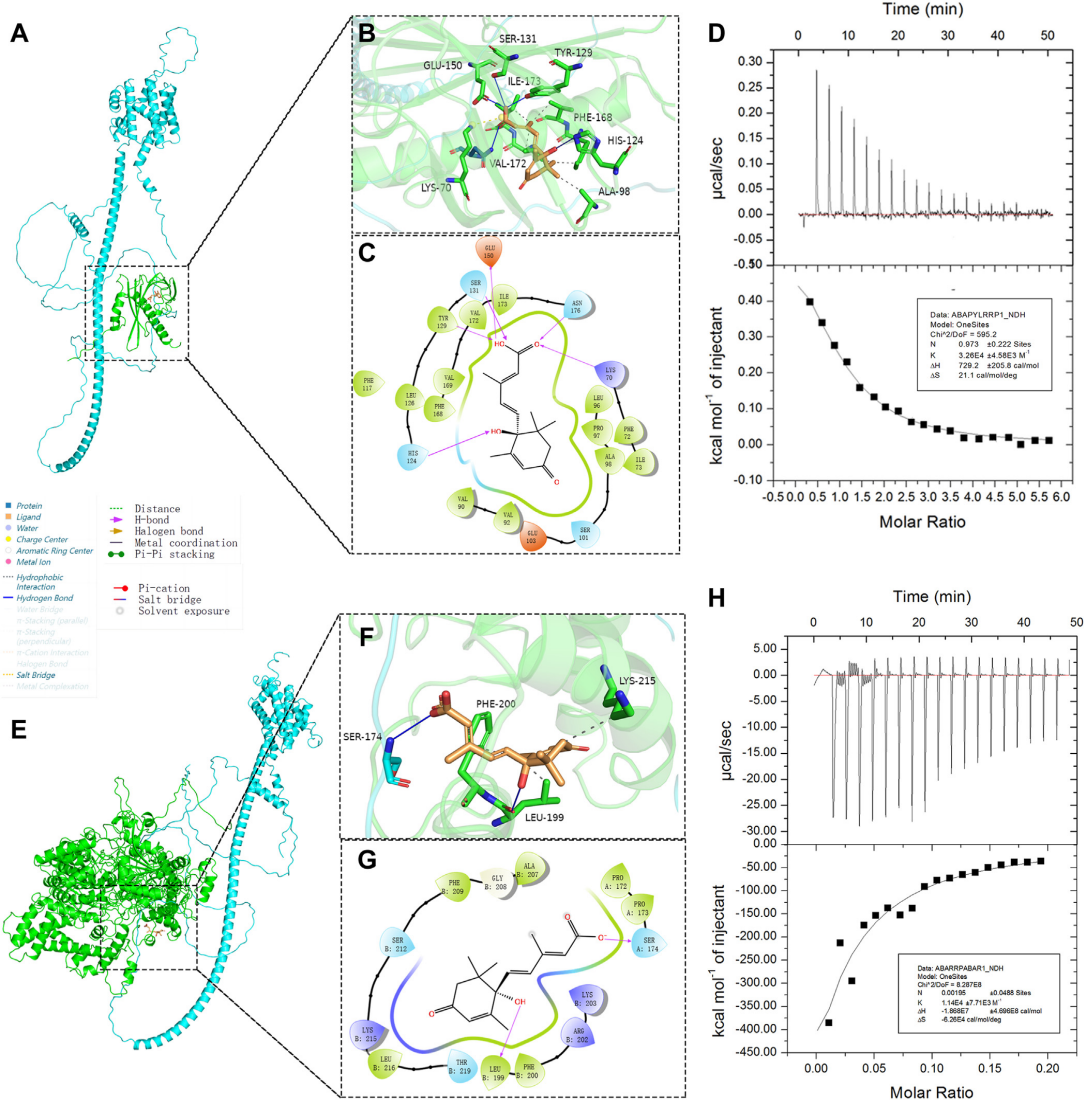
**FaRRP1–FaPYL2 and FaRRP1–FaABAR complexes bind to ABA.***A–C* and *E–G*, using Schrödinger to analyze the ABA and FaRRP1-FaPYL2 and FaRRP1-FaABAR protein complex 3D structures and the Glide module for protein-ABA semiflexible docking. It was plotted in pyMOL. *D*, 0.4 mM ABA titrate 20 μM FaRRP1–FaPYL2 protein complexes solution. *H*, 0.4 mM ABA titrate 20 μM FaRRP1–FaABAR protein complexes solution. Data are shown as the mean ± standard error (n = 3). ABA, abscisic acid.

Although FaRRP1 interacts with FaPYL2 and FaABAR, the interacting significance remains unknown. Given that they all bind to ABA (Fig. 1), we explore the relationship of ABA with the pockets of FaRRP1–FaPYL2 and FaRRP1–FaABAR complexes by a series of *silico* analyses, including the Schrödinger for predicting the ABA and protein 3D structures, the Glide module for detecting protein-ABA semiflexible docking, and the SiteMap for investigation of predicted docking pockets and SP-docking. Finally, the highest-scoring conformation was selected for analysis of each docked small molecule, causally associated to a conformation. The results showed that the active pocket of FaRRP1–FaPYL2 protein complex binding to ABA was located in the FaPYL2 polypeptide chains: Lys-70 to Ile-73, Leu-96 to Glu-103, His-124 to Ser-131, and Phe-168 to Asn-176 (Fig. 5, *A*–*C*). By contrast, the active pocket of FaRRP1-FaABAR binding to ABA was located at the site of FaRRP1–FaABAR interaction: Pro-172 to Ser174 of FaRRP1 and Leu-199 to Thr-219 of FaABAR (Fig. 5, *E*–*G*). These analyses suggest that the FaRRP1-FaPYL2 and FaRRP1-FaABAR complexes may bind to ABA.

Whether FaRRP1 affects the binding activity of FaPYL2 and FaABAR to ABA is unclear. For this, FaRRP1-His/MBP, FaPYL2-His, and FaABAR-His were purified and dialyzed into the same buffer (ITC Buffer) (20 mM Na_2_HPO_4_, 20 mM NaH_2_PO_4_, 300 mM NaCl, 20 mM MgCl_2_ pH 7.4). Then, the molar concentrations of FaRRP1-FaPYL2 and FaRRP1-FaABAR complexes were mixed into the same ratio and placed at 4 °C for 30 min. Finally, 1-fold protein peptide and 20-fold S- (+)-ABA were used for ITC assays (Fig. 5, *D* and *H*). The results showed that the binding of FaRRP1–FaPYL2 complex to ABA followed an endothermic reaction with a Kd of 30.6 μM (Fig. 5*D*) and that of FaRRP1–FaABAR complex to ABA was an exothermic reaction with a Kd of 87.7 μM (Fig. 5*H*). These results demonstrate that FaRRP1 may affect the binding affinity of ABA to both FaPYL2 and FaABAR.

### FaRRP1, FaPYL2, and FaABAR positively regulate strawberry fruit ripening

**Figure 6:**
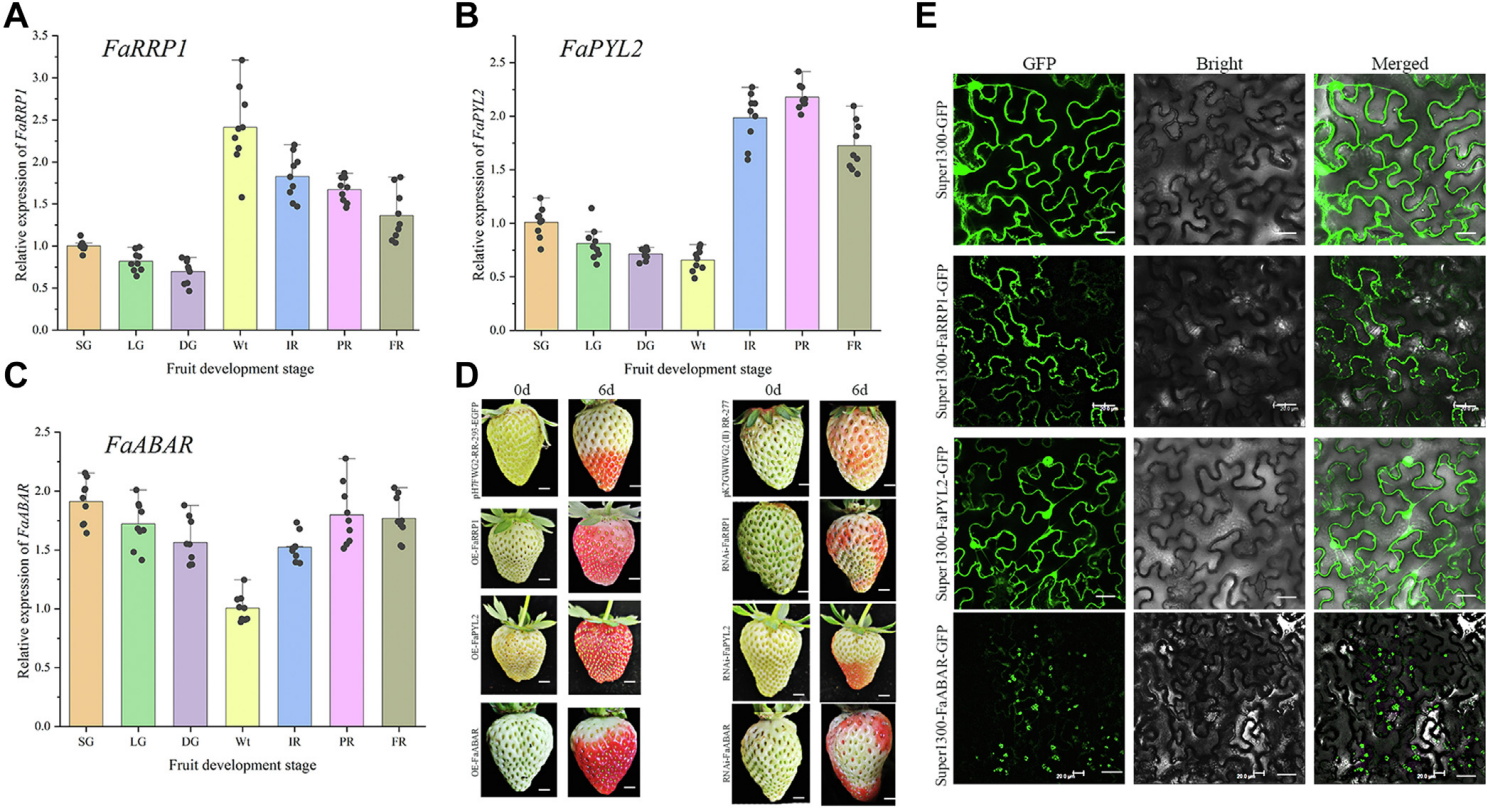
**Functional analysis of *FaRRP1, FaPYL2, and FaABAR* genes.***A–C*, expression patterns of *FaRRP1, FaPYL2, and FaABAR* genes in ‘Monterey’ strawberry. The developmental processes of Monterey fruit were divided into seven stages, namely, small green (SG), large green (LG), de-green (DG), white (Wt), initial red (IR), partial red (PR), and whole red (FR) stages. The experiment was performed with three replicates. *D*, *Agrobacterium* GV3101 strains containing RNAi (intron–hairpin RNA interference) or overexpression *FaRRP1, FaPYL2, and FaABAR* recombinant plasmids were injected into DG fruits attached to the plant. The fruit phenotype was recorded 6 days after injection. pK7GWIWG2 (II) RR-277 and pH7FWG2-R3-EGFP were used as RNAi and OE control groups. The experiment was performed with three replicates. Bars represent 1 cm. *E*, FaRRP1-GFP transiently expressed in *Nicotiana benthamiana* leaves was localized to cytoplasmic membrane and clathrin; FaPYL2-GFP is localized in the cytoplasm and FaABAR-GFP in the chloroplast and nucleus. The experiment was performed with three replicates. Bars represent 20 μm.

Given FaRRP1, FaPYL2, and FaABAR, as described above, all are associated to ABA, which is critical to strawberry fruit ripening (7, 10), thus we subsequently investigate the three protein functions in ripening. First, quantitative reverse transcription PCR (RT-qPCR) was performed to analyze the expression levels of *FaRRP1, FaPYL2*, and *FaABAR* using the complementary DNA (cDNA) from seven-stage (SG, small green; LG, large green; DG, de-greening; Wt, white; IR, initial red; PR, partial red; FR, full red) fruits as application templates (Fig. 6, *A*–*C*). The results showed that the expression of *FaRRP1* and *FaPYL2* increased and maintained high levels during ripening from IR to FR stages (Fig. 6, *A* and *B*). In contrast, the expression of *FaABAR*, on the whole, maintained stable levels, in addition to an increase during onset-ripening from Wt to PR stages (Fig. 6*C*). These data suggest that FaRRP1, FaPYL2, and FaABAR may function in fruit ripening.

Second, to further determine these gene functions, the constructed FaRRP1-RNAi/overexpression (OE), FaPYL2-RNAi/OE, FaABAR-RNAi/OE (RNA interference/overexpression) vectors were transferred into *Agrobacterium* GV3101 (WEIDI). Strawberry fruits were infected at the DG stage and observed after 6 days. The results showed that the OE vector–treated strawberry fruits were in the FR stage, and the control strawberry fruits were in the IR stage, while the RNAi vector–treated fruits showed a chimeric phenotype (Fig. 6*D*), demonstrating that FaRRP1, FaPYL2, and FaABAR participate in the coloring and ripening of strawberry fruits as positive regulators. Notably, although *FaABAR* expression changed a little in the developmental fruit (Fig. 6*C*), the manipulation of the gene expression affected the ripening (Fig. 6*D*), consistent with the previous reports (31, 34). Therefore, the in-depth mechanism of FaABAR in ripening needs to be explored.

**Supplemental Fig. S6:**
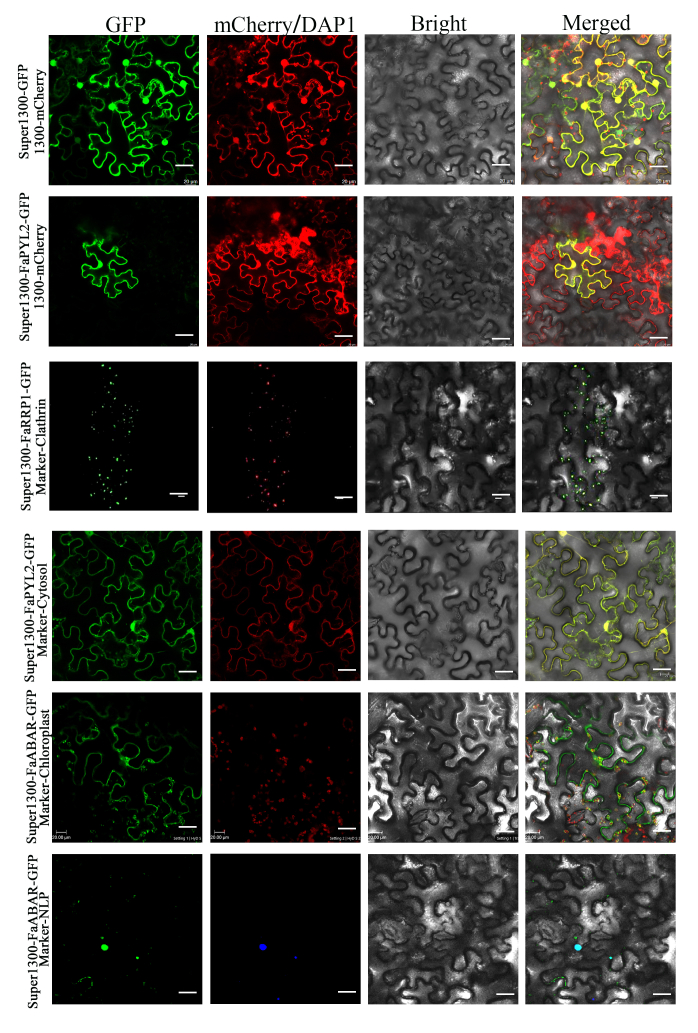
**Observation of FaRRP1, FaPYL2, and FaABAR subcellular fraction in lower epidermal cells of *Nicotiana benthamiana* leaves under the laser confocal microscope.** Bars: 20 μm. Among them, Super1300-GFP and 1300-Mcherry, Super1300-FaPYL2 and 1300-Mcherry are the control group; Marker-Clarthin and FaRRP1-GFP have overlapping fluorescence; Marker-Cytoplasmic and FaPYL2-GFP have overlapping fluorescence; Marker-Chloroplast and FaABAR-GFP have overlapping fluorescence; FaABAR-GFP and DAP1 (4',6-diamidino-2-phenylindole) showed overlapping fluorescence after staining with 10 μM DAP1.

To understand their roles, we have constructed FaRRP1-GFP, FaPYL2-GFP, and FaABAR-GFP recombinant plasmids, which were transformed into *Agrobacterium* GV3101 for infecting lower epidermal cells of *N. benthamiana* leaves. Under the laser confocal microscope, we found that FaRRP1 was located on the cytoplasmic membrane and clathrin; FaPYL2 was localized in the cytoplasm; and FaABAR was localized in the chloroplast and nucleus (Fig. 6*E*), by contrast to their corresponding marker–localized molecules (30, 35, 36) (Fig. S6). Given FaRRP1 and FaABAR can interact (Fig. 4, *A*–*C*) but with different subcellular localizations (Figs. 6*E* and S6), as well as on the basis of the previous reports (15, 31, 37), we postulate that FaABAR may migrate among chloroplast, nucleus, and cytoplasm under certain developmental and environmental conditions, permitting them to interact potentially by FaRRP1-mediated endocytosis and vesicle transport to facilitate FaPYL2 and FaABAR signaling. Altogether, FaRRP1, FaPYL2, and FaABAR take part in the initiation of strawberry fruit ripening, potentially by FaRRP1-mediated ABA trafficking and signaling.

### Transient RNAi of FaRRP1 and ABA treatment could induce clathrin-mediated vesicle transport evidenced by a line of relative gene expression

**Figure 7:**
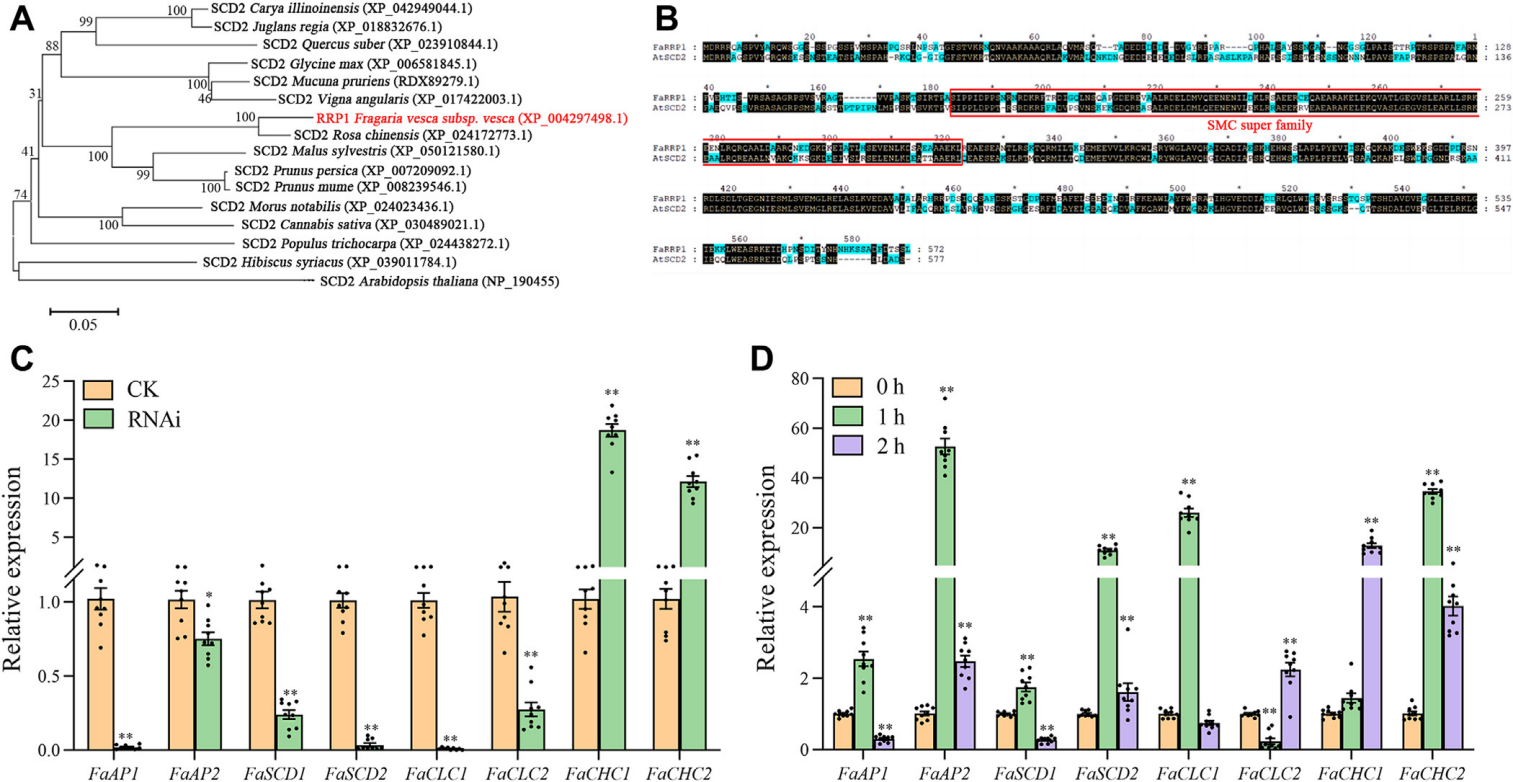
**Bioinformatics and fluorescence RT-qPCR analysis FaRRP1 relationship with clathrin-mediated vesicle transport.***A*, phylogenetic tree of the FaRRP1 protein with its homologous proteins from other plant species. The branch lengths are proportional to distance. *B*, sequence alignment of the FaRRP1 protein with its homologous proteins AtSCD2. *Red* boxes mark the domain of SMC (structural maintenance of chromosomes). *C*, transcript levels of clathrin-mediated vesicle transport genes detected by real-time PCR. *FaActin* mRNA was used as an internal control. *D*, relationship of ABA with clathrin-mediated vesicle transport genes’ transcription-quantitative PCR analysis of genes expression in response to 100 μM ABA. The experiment was performed with three replicates. Asterisks indicate significant differences from the control (paired test): ∗*p* <0.05; ∗∗*p* <0.01. ABA, abscisic acid; FaAP1, adapter protein complex 1; FaAP2, adapter protein complex 2; FaCHC1, clathrin heavy chain 1; FaCHC2, clathrin heavy chain 2; FaCLC1, clathrin light chain 1; FaCLC2, clathrin light chain 2; FaSCD1, stomatal cytokinesis defective 1; FaSCD2, stomatal cytokinesis defective 2; RNAi, RNA interference.

To explore whether FaRRP1 regulates the ripening in relation to clathrin-mediated vesicle transport, first, the amino acid sequences of FaRRP1 and its 16 homologs from various species were compared, then a phylogenetic tree was constructed using the MEGA5.0 software (http://www.megasoftware.net/) (Fig. 7*A*). The results showed that FaRRP1 had the highest homology with *Rosa chinensis* SCD2 (XP_024172773.1) and possessed a close genetic relationship with *Malus sylvestris* SCD2 (XP_050121580.1), *Prunus persica* SCD2 (XP_007209092.1), *Prunus mume* SCD2 (XP_008239546.1) in the same clade with relatively high homologies (Fig. 7*A*). Thus, FaRRP1 is a homologous gene of SCD2. Subsequently, the amino acid multiple sequence alignments of FaRRP1 and AtSCD2 (29) were performed using GeneDoc software (https://genedoc.software.informer.com/) (Fig. 7*B*). It was found that FaRRP1 and AtSCD2 proteins had a highly conserved structural maintenance of chromosomes region superfamily, which contained about 140 amino acid residues (Fig. 7*B*). In addition, we used AlphaFold2 v2.3 to predict the 3D structure of FaRRP1 protein and found the highest homology with the 3D design of AtSCD2 (Fig. S3*A*). These data indicate that FaRRP1 may have similar function of SCD2.

Notably, the SCD2 protein was previously found to be associated with clathrin-coated vesicles and colocalized with the clathrin light chain at putative sites of endocytosis at the PM (29, 35). Therefore, we selected a series of genes involved in clathrin-mediated endocytosis, including clathrin heavy chains (*FaCHC1* and *FaCHC2*), clathrin light chains (*FaCLC1* and *FaCLC2*), adapter protein complex (*FaAP1* and *FaAP2*), stomatal cytokinesis defective (*FaSCD1* and *FaSCD2*), which were used for gene expression analyses in transient RNAi of *FaRRP1* and ABA-treated strawberry fruits (Fig. 7, *C* and *D*). Interestingly, the expressions of *FaAP1, FaAP2, FaSCD1, FaSCD2, FaCLC1*, and *FaCLC2* were significantly inhibited in the RNAi fruit (Fig. 7*C*), while the expressions of *FaCHC1* and *FaCHC2* were upregulated considerably (Fig. 7*D*). These results suggest that manipulating *FaRRP1* expression may inhibit the expression of *FaSCD1* and *FaSCD2*, as well as *FaCLC1* and *FaCLC2*. As a result, this led to *FaCHC1* and *FaCHC2* essential to the endocytosis (Fig. 7*C*). After 100 μM ABA treatment for 1 h, the expressions of *FaAP1, FaAP2, FaSCD1, FaSCD2, FaCLC1*, and *FaCHC2* were significantly increased, while the expressions of *FaCLC2* were decreased considerably, but the expressions of *FaCHC1* were not changed. After ABA treatment for 2 h, the results showed that compared with the control, the expressions of *FaAP1, FaSCD1*, and *FaCHC2* genes were significantly decreased, the expressions of *FaCLC2* and *FaCHC1* genes were quite upregulated, and the expressions of *FaAP2* and *FaSCD2* genes were significantly increased but significantly decreased in contrast to the 1-h-treatment control (Fig. 7*D*). These results suggest that the clathrin-related genes may be induced by ABA, and FaRRP1 could transport ABA through clathrin-mediated endocytosis.

### FaRRP1-mediated cellular ABA uptake by clathrin-mediated endocytosis

**Figure 8:**
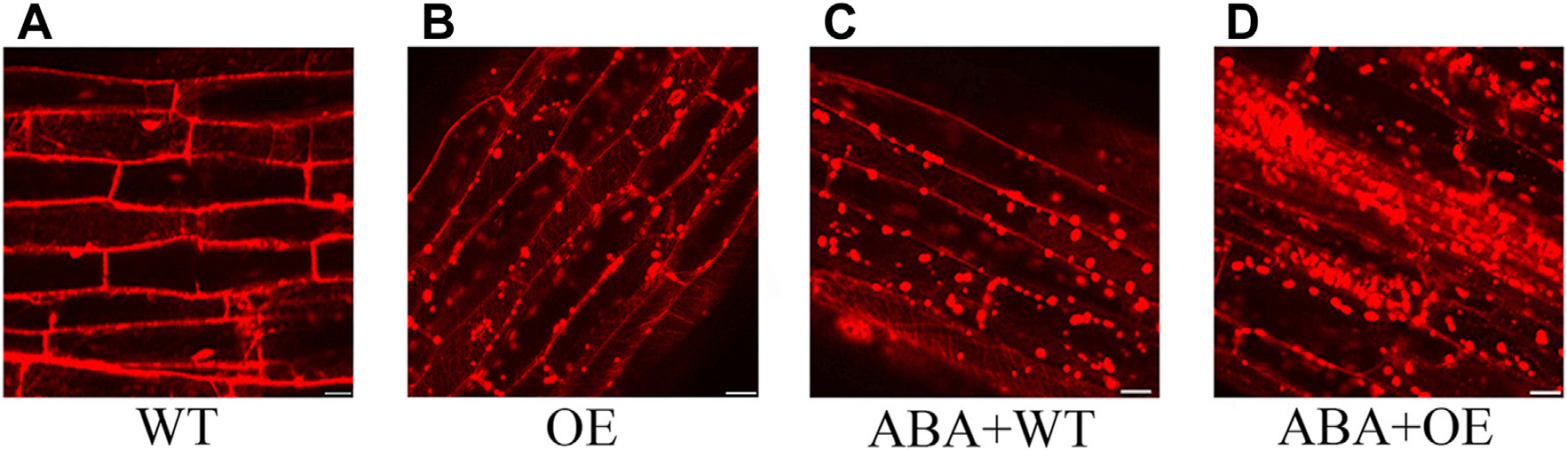
**ABA promotion of FaRRP1-mediated endocytosis.***A–D*, the 10-days-old WT and FaRRP1-OE seedlings were treated with 0.1 μM ABA for 48 h and stained with 5 μM FM4-64 dye for 10 min. Bars represent 20 μm. ABA, abscisic acid.

The data above include that the manipulation of *FaRRP1* expression (Fig. 7*C*) and ABA treatment (Fig. 7*D*) could modulate the clathrin-related marker gene expression and that FaRRP1 was localized to the cytoplasmic membrane (Fig. 6*E*) and clathrin (Fig. S6), evoking us to investigate a direct relationship of FaRRP1-mediated endocytosis with ABA using the combination of the root system of *RRP1*-transgenic Arabidopsis reported by McMichael *et al.* (29) and exogenous ABA application. Namely, the WT and FaRRP1-OE plants were treated with exogenous ABA, and then the lipophilic dye FM4-64 (20) was used to determine the cellular internalization. Then the WT and FaRRP1-OE transgenic Arabidopsis roots were laboratory-retained for imaging by confocal laser scanning microscope (Fig. 8*A*). The results showed that the overexpression of *FaRRP1* (Fig. 8*B*) showed an increase in FM4-64–labeled fluorescent signal in root cells, and exogenous ABA could promote the degree of PM internalization in the FaRRP1-OE line than the WT (Fig. 8, *C* and *D*). These data prove that ABA promotes FaRRP1-mediated endocytosis and vesicle transport.

## Discussion

In higher plants, a vital developmental phase is the transition of vegetative-to-reproductive growth for the production of fruits. At cellular levels, fleshy-fruit development undergoes in turn two vital processes from early cell division to the cell expansion, which is driven by sugar accumulation and metabolism in vacuoles, a major part of pulp cell volume. To this end, FaRRP1-mediated vesicular transport may contribute to our better understanding of the osmotic-driven vacuole expansion determined by high accumulation of sugars and metabolites, essential to fruit growth and ripening.

### FaRRP1 is first demonstrated to positively regulate strawberry fruit ripening by clathrin-mediated vesicle transport

FaRRP1 is the previously identified homolog of the SCD2 protein containing a coiled-coil structure, sharing the same domain with SCD2 (Fig. 7), key to clathrin-mediated vesicle transport involved in various biological processes (23, 24). In the present study, we first find that FaRRP1 has the highest expression in the white strawberry fruit (Fig. 6*A*), suggesting its function in the initiation of ripening. Notably, the coiled-coil domain is one of the most abundant interacting motifs essential to multiple biological functions such as transcription, signaling, and protein folding (38). Consistently, the previous and our data indicate that FaRRP1/SCD2 is a member of a small family of plant-specific genes, which is localized to the cytoplasmic membrane and colocalizes with clathrin light chain 2 (CLC2) (Fig. S6), associated to plant growth and development by the control of cell division and expansion in a dependence of clathrin-mediated endocytosis (29, 30, 31) (Fig. 6*E*). Altogether, we now have provided a line of data to demonstrate a new role of FaRRP1/SCD2 in the positive regulation of strawberry fruit ripening by the clathrin-mediated vesicle transport (Figs. 7 and 8), to our knowledge.

### FaRRP1 regulates strawberry fruit ripening by clathrin-mediated integration of ABA trafficking and signaling

ABA is a vital regulator important to strawberry fruit ripening and quality (8). Under normal conditions, a considerable part of ABA is free to diffuse (34, 35), while under stressful situations, ABA moves into cells, mediated by its transporters in a vesical trafficking manner (36, 37, 38, 39). Thus, this evokes us to explore the mechanism of ABA in clathrin-mediated vesical transport by FaRRP1/SCD2.

It is known that clathrin-mediated endocytosis is the most characteristic endocytic pathway, associated to cell surface protein internalization in response to extracellular and intracellular signals (39). The clathrin is a tripod structure consisting of three light chains of CLC and three heavy chains of clathrin heavy chain (CHC) (40). Also, previous studies have shown the significant role of CHC functions in intracellular trafficking (39, 41) by affecting the trimerization and stability of the CHC (42, 43), which is enhanced by the C-terminal domain of the light chain (44). Confirmedly, by a combination of the manipulation of *FaRRP1* expression and ABA treatment (Fig. 7), we find a role of FaRRP1 in the regulation of the mRNA levels of several clathrin-mediated endocytosis-associated genes (*FaAP1*, *FaAP2, FaCLC1, FaCLC2, FaSCD1, FaSCD2*, *FaCHC1*, and *FaCHC2*). Notably, the mRNA levels of *FaCHC1* and *FaCHC2* were significantly increased in transient RNAi fruit of *FaRRP1*; maybe this increase could be as a result of *FaRRP1* downregulation-mediated decrease in the mRNA levels of *CLCs* of *CHCs* (Fig. 7) to compensate for the CLC-function lacking, suggesting that FaRRP1 may function mainly by CLCs.

Indeed, the light chain connects the endocytic machinery and the cytoskeleton (45) and plays an essential regulatory role in cage assembly (44, 46, 47). In addition, the clathrin light chains also play an important role in plant development, such as the loss of CLC2 and CLC3, which affect auxin-regulated endocytosis, resulting in multiple developmental defects in *A. thaliana* (48). Also, the knockout of *CLC2* and *CLC3* causes hypocotyl elongation in Arabidopsis (49), associated with the signaling pathways of both brassinosteroid (50) and ABA (30, 36). The present study finds that FaRRP1 is localized in the cytoplasmic membrane, binds to ABA, and functions in the ripening (Figs. 1 and 7). Moreover, we determine the same function of FaRRP1 with AtSCD2 functioned in endocytosis and membrane transport (Fig. 7), which is facilitated by the ABA promotion of FaRRP1-mediated process (Fig. 8). By the combination of previous reports (36, 51) and the present results (Figs. 1, 7, 8, and S6), it is strongly suggested that FaRRP1 may be involved in ABA movement.

Protein construction is the basis of its biological function (32); strikingly, Schrödinger has been used extensively, especially in molecular docking or virtual screening (52, 53). FaPYL2 and FaABAR are previously reported to be candidate receptors for ABA (24, 54), and now together with FaRRP1, they all have been shown to bind to ABA (Fig. 1). On the basis of Schrödinger simulations, we find the positioning of substrates (ABA) with catalytic pockets in FaRRP1, FaPYL2, and FaABAR for ABA binding (Fig. 1). Notably, FaRRP1, FaPYL2, and FaABAR form hydrogen bonds and ionic interactions with the carboxyl (-COOH) and hydroxyl (-OH) groups of ABA, but not with the carbonyl (C=O) group of ABA (Fig. 1). Confirmedly, similar results were also shown at the ABA-binding sites of FaRRP1–FaPYL2 and FaRRP1–FaABAR complexes (Fig. 5). As an electron-withdrawing group, although the carbonyl group (C=O) would make the hydrogen on its adjacent atoms exhibit considerable activity and dissociate more easily (55, 56, 57), the carbonyl (C=O) group has been used to prepare biologically active ABA-photoaffinity labels (58, 59). In summary, the process of the ABA–protein interaction in the binding pocket is mainly dependent on the carboxyl (-COOH) and hydroxyl (-OH) groups of ABA, rather than its carbonyl group (C=O).

Notably, the crystal structure of Arabidopsis PYL2 (AtPYL2) protein and its ligand-binding pocket to ABA have been resolved (22, 25, 60). The specificity of PYL2 for ABA recognition is mainly due to two charge-stabilized hydrogen bonds between the carboxylate of ABA and Lys-64 of PYL2 (25, 60), which are consistent with our predicted ligand-binding pockets (Fig. 1). Namely, we found charge-stable hydrogen bonds between Lys-70 of FaPYL2 and the carboxylate of ABA (Fig. 1). It is previously reported that ABA directly binds AtPYL2 by the sites of Lys-64, Phe-66, Val-87, Ala-93, His-119, Leu-121, Tyr-124, Val-166, and Val-169 (60), which are similar to the ABA-binding pocket of FaPYL2, including Lys-70, Ile-73, Val-92, Ala-98, Ser-101, Glu-103, Ser-131, Glu-150, Val-172, Ile-173, and Asn-176 (Fig. S4). These data suggest that although the PYL2 exists differences at the amino acid level between various species, the PYL2-mediated ABA perception mechanism might be conserved, providing a new insight into the molecular mechanism of FaPYL2-triggered ABA signaling in strawberry fruit ripening.

Similarly, the structure of prokaryote ABAR has been resolved (61, 62, 63, 64), and Lys-950 to Val-1330 was found to be the active site region in binding to the porphyrin IX (64). Notably, the residues of FaABAR for ABA recognition are mainly due to charge-stabilized hydrogen bonds between the hydroxyl of ABA and Asp-1091 and Ser-1146 of FaABAR (Fig. 1), consistent with a previous report at the C-terminal binding (10). Interestingly, we found that FaRRP1 interacts with FaPYL2 and FaABAR (Fig. 4), and the FaRRP1–FaABAR interaction altered the location of the binding pocket with ABA; in contrast, FaRRP1–FaPYL2 interaction did not alter the ligand-binding pocket site (Fig. 5), suggesting a complexity of FaABAR in binding to ABA. After the combinational gene localization and expression analyses (Figs. 6 and 7), we consider that FaRRP1–FaPYL2 interaction may play a central role in the regulation of strawberry fruit ripening.

Consistent with a previous report that FaPYR1, as a member of FaPYR/PYL families, positively regulates strawberry fruit ripening (9), FaPYL2 not only has top expression during the ripening (11) (Fig. 6) but also interacts with FaABI1, a core component in ABA signaling (12). In addition, it also confirmed the role of ABAR in the ripening of strawberry (11, 15) (Fig. 6) and peach (65, 66). These notions are not only confirmed in the present study but we also find that FaRRP1 binds to ABA and interacts with both FaPYL2 and FaABAR, and the interaction may affect their perception affinity to ABA (Figure 1, Figure 2, Figure 3, Figure 4, Figure 5, Figure 6, Figure 7, Figure 8). Altogether, FaRRP1 regulates strawberry fruit ripening by the integration of ABA trafficking into the two signaling pathways of FaPYL2 and FaABAR, contributing to the fine-tuning control of ripening processes.

In addition, it is also notable that although the stoichiometric (N) value may be gained by ITC assay, our gained N values showed considerable differences by ITC. For example, FaRRP1, FaPYL2, and FaABAR bound to ABA with the N data were 0.41, 2.63, and 8.60, respectively (Fig. 1). Maybe, these differences might be due to several reasons: (1) limitations of the *Escherichia coli* expression system (67). Although the *E. coli* expression system is widely used in foreign protein expression, the lack of posttranslational modification affects the protein activity in binding analyses; (2) protein natural properties also influence the N data in ligand-binding experiments. Such as, the theoretical instability coefficient of FaRRP1 protein had the high half-life period in the *E. coli* cells during induced expression, showing an unstable nature. Notably, PYL2 exists both monomers and dimers, which showed the different binding affinity to ABA (20, 25), such as the dimer PYL1 with Kd values at 52 to 340 μM (26, 68); maybe, this is similar to FaPYL2 with the alterable N data (Fig. 1). Also, it is not only noticed that the oxidation may induce the oligomerization of ABAR (69) but also this 160-kDa recombinant purified protein is not easy to control the protein concentration, purity, and activity, mostly leading to the significant difference in binding experiments. Altogether, some various uncontrollable factors in ITC assay should lead to the N values with great differences.

### A proposed model for the molecular mechanism of FaRRP1 in regulation of strawberry fruit ripening by ABA

In the present study, we find that FaABAR is localized in chloroplasts and the nucleus (Fig. S6), supported by previous studies (15, 16, 70), suggesting that FaABAR may be widely involved in plant growth and development by various mechanisms. To some extent, our results show that FaABAR and FaRRP1 interact in the PM by BiFC assays (Fig. 4), potentially providing a theoretical basis for FaABAR to transport from chloroplast into the nucleus. Notably, a strawberry leu-rich repeat receptor-like kinase, red-initial protein kinase 1 (FaRIPK1), is demonstrated to interact with FaABAR and positively regulates the fruit ripening, suggesting a synergistic action of FaRIPK1 with FaABAR in the ripening (15). Also, a recent report finds that FvRIPK1 interacts with FvSnRK2.6 and phosphorylates each other (16), supporting a link between ABAR and SnRK2.6 in guard cell signaling in response to ABA (18) and a proposed ABA-ABAR-RIPK1-SnRK2.6-ABI4 model in strawberry fruit ripening (7, 8), which are further clarified by the present study.

**Figure 9:**
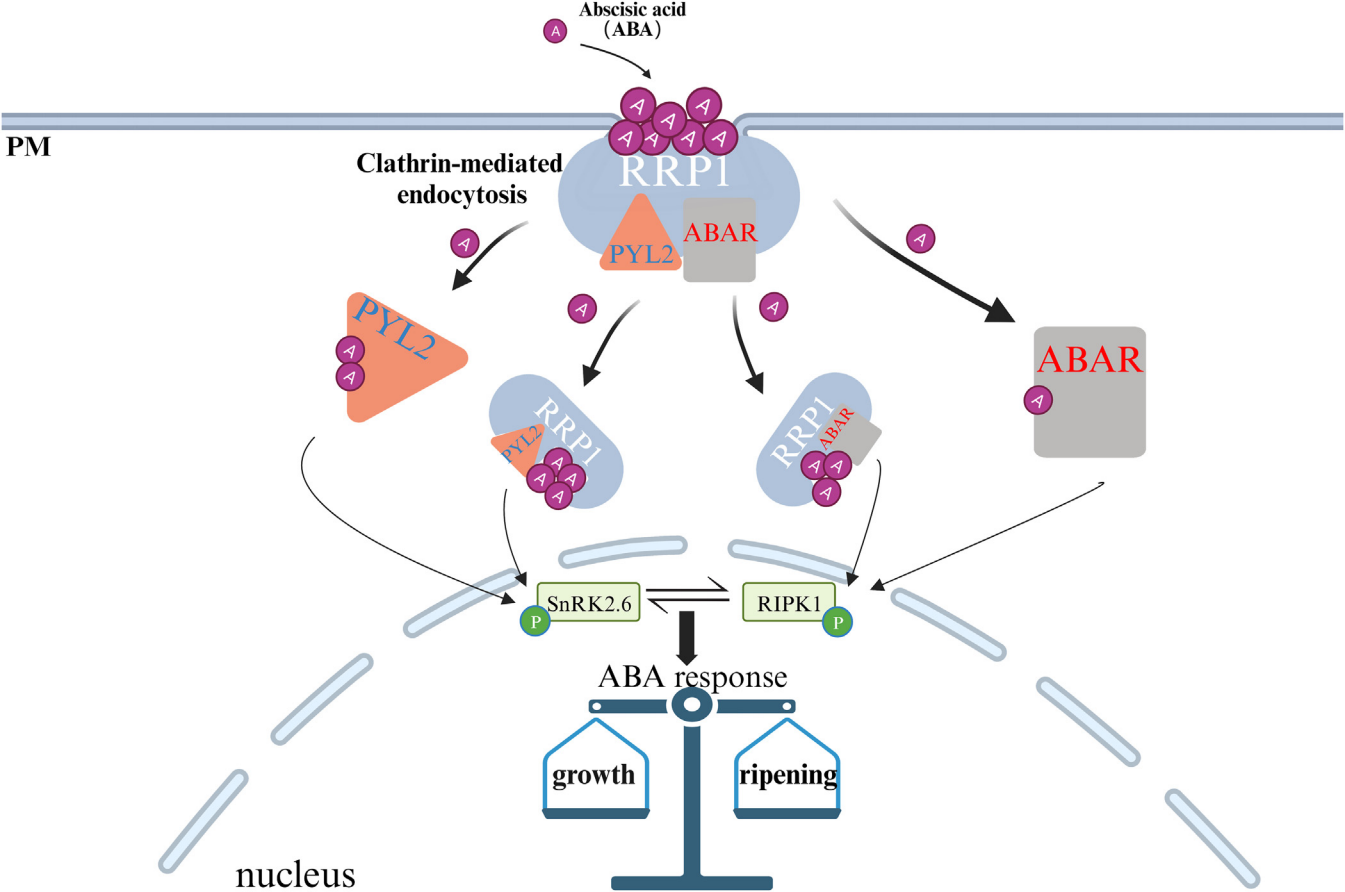
**A model for RRP1-mediated endocytosis of ABA and ABA signaling.** The RRP1 binds ABA in the plasma membrane and subsequently the entry into cells, enhancing the binding affinity of PYL2 and ABAR to ABA, ultimately promoting ABA response and fruit ripening. ABA, abscisic acid.

The facts including the critical roles of both RRP1/SCD2 in cytokinesis and cell expansion (22) and the osmotic-driven cell expansion in fruit development (71), as well as ABA signaling in fruit ripening (8) and our present results (Figure 1, Figure 2, Figure 3, Figure 4, Figure 5, Figure 6, Figure 7, Figure 8), evoke us to propose a model for multiple ABA signaling pathways linked to strawberry growth and fruit ripening potentially by FaRRP1-mediated ABA trafficking and signaling (Fig. 9). The RRP1 binds ABA at the PM to initiate clathrin-mediated endocytosis of ABA into cells, subsequently facilitating ABA binding to PYL2/ABAR, then relaying two ‘ABA-PYL2-ABI-SnRK2.6’ and ‘ABA-ABAR-RIPK1-SnRK2.6’ signaling pathways for synergetic control of strawberry fruit ripening. Of course, more visual and molecular evidence is to be provided at tissue, cellular, subcellular, and structural levels for our further better understanding of the proposed model in plant growth and development as well as fruit ripening under various environmental conditions.

## Experimental procedures

### Plant material and growth conditions

The experimental material used in this study was octoploid strawberry (*Fragaria × ananassa* ‘Monterey’) planted at the Beijing University of Agriculture. Grow in a natural light greenhouse with a relative humidity of 60%-75% with a 14-h day (25 °C ± 2 deg. C)/10-h night (10 °C ± 2 deg. C) cycle. Samples were collected at seven stages of strawberry fruit development and stored at −80 °C for use.

### RNA isolation and cDNA synthesis

Total RNA was separately isolated from the strawberry tissue of fruits in the Total RNA Extraction Kit (Magen) by the manufacturer's protocols. The purity and integrity of the RNA were determined by gel electrophoresis and the ratios of A260/A230 and A260/A280. To generate first-strand cDNA, 400 ng of total RNA was reverse transcribed using the HifairⅡ 1st Strand cDNA Synthesis Kit (gDNA digester plus) (Yeasen) by the manufacturer's protocols.

### AlphaFold protein prediction and figure generation

To construct a 3D model structure of FaRRP1, FaPYL2, and FaABAR, their amino acid sequences were obtained in the FASTA format from the NCBI database (https://www.ncbi.nlm.nih.gov/) (72). Subsequently, each sequence was provided to the AlphaFold2 v2.3 program for structure generation (32). The three-dimensional coordinate files were extracted from the ProteinDataBank. The predicted first- to fifth-ranked models pLDDT and the PAE plot are in the supplementary material (Fig. S1). We usually score the first ranked model the model/rank 1 model.

### Receptor preparation and grid generation

An essential step for docking/virtual screening is receptor preparation. The preparation of ABA receptors includes assigning bond orders and adding missing hydrogen atoms, followed by optimizing orientations of hydrogen-bonded groups and minimizing the structure using optimized potentials for liquid simulations (OPLS-3) force field (73). Protein preparation is carried out using the Protein Preparation Wizard module of Schrödinger software, including assignment of bond levels, hydrogenation, etc., removal of water molecules and cofactors from the protein, optimization of the hydrogen bonding network, and finally protein energy minimization using the OPLS_3e force field. This process was performed using the Protein preparation wizard module in Schrödinger (74, 75). Afterward, the receptor grid box was generated using the receptor grid generation wizard in the Glide grid module in Schrödinger, docking using SiteMap predictive docking pockets with SP-docking. We use Schrödinger's built-in GlideScore as a scoring function. For each docking simulation, 10 conformations were generated for each receptor, and they were docked for postdocking energy minimization. The highest score was selected for subsequent analysis for each conformation generated by each docked receptor.

### Protein-protein docking

Protein preparation was carried out using the Protein Preparation Wizard module of the Schrödinger software. The two proteins are first assigned bond levels, hydrogenated, assigned zero-level bonds to metal atoms, and disulfide bonds are created separately, followed by optimization of the hydrogen bonding network and finally protein energy minimization using the OPLS_3e force field. Molecular docking is performed using the Protein-Protein docking (Piper) module in Schrödinger. Docking was performed using the standard mode, with the number of rotatable probes for the ligand defined as 70,000, which allowed for adequate conformational sampling of the ligand-protein, and the number of conformations generated was defined as 30. For proteins with multiple chains, we used only one chain for docking. Piper clustered the first 1000 rotated conformations of all the generated conformations based on the RMSD between each atom, with the representative conformation in each class being selected from the conformation with the most neighbors in that class. Piper ranked the generated conformations based on the number of clusters in each class, with the first ranked conformation having the highest number of clusters, and we selected the first conformation for subsequent analysis.

### Expression, purification, and identification of FaRRP1, FaPYL2, and FaABAR

The expression and purification of the recombinant proteins FaRRP1, FaPYL2, and FaABAR were done by a prokaryotic (*E. coli*) expression system. The cDNA encoding the FaRRP1, FaPYL2, and FaABAR was synthesized using the aforementioned method by PCR amplification using the primers listed in Table S1. The recombinant plasmids of pET-28a-FaPYL2-His, pMAL-C5X-FaRRP1-His/MBP, pGEX4T-1-FaRRP1-GST, pET-28a-FaABAR-His were constructed. After positive recombinants were identified by double-digesting and sequencing, the *E. coli* strain BL21 (DE3) cells with the recombinant plasmid were grown in 500 ml LB medium containing different antibiotics at 37 °C. On A_600_ at 0.6 to 0.7, 0.5 mM IPTG was added to inducibly express the fusion proteins overnight at 16 °C for 12 h. The cultures were harvested and suspended with 20 ml binding buffer (20 mM Na_2_HPO_4_, 20 mM NaH_2_PO_4_, 300 mM NaCl, 20 mM MgCl_2_, 10 mM imidazole, pH 7.4), then lysed by sonication in a Misonix Sonifier at 4 °C; finally, the supernatants were loaded onto BeaverBeads IDA- Ni^2+^ (Beaver) columns and mixed gently by shaking at 4 °C for 60 min. The lysate-beads mixture was loaded into an empty column and separated by the magnetic separator. The beads binding destined protein was washed with washing buffer (20 mM Na_2_HPO_4_, 20 mM NaH_2_PO_4_, 300 mM NaCl, 20 mM MgCl_2_, 50 mM imidazole, pH 7.4) and eluted with elution buffer (20 mM Na_2_HPO_4_, 20 mM NaH_2_PO_4_, 300 mM NaCl, 20 mM MgCl_2_, 350 mM imidazole, pH 7.4) for 2 ml.

### Measurement of protein adsorption by the BCA method

We used the BCA assay (74) to determine the purified protein concentration. The experiment was done as described by Olson BJ *et al*. (76). From the standard curves, protein concentration (μg/μl) was calculated. The experiment was performed with three replicates.

### ITC assay

The purified protein was concentrated using an Amicon Ultra-4 centrifugal 3-kDa filter (Millipore) at 3000*g* for 20 min in a swing bucket rotor (Sigma). The protein was then desalted for buffer exchange using a HiTrap desalting column (GE Healthcare). The HiTrap desalting column was filled with ITC buffer (20 mM Na_2_HPO_4_, 20 mM NaH_2_PO_4_, 300 mM NaCl, 20 mM MgCl_2_, pH 7.4) to remove ethanol completely and equilibrate the column. The final concentration of the FaRRP1-His/MBP, FaPYL2-His, and FaABAR-His fusion protein was adjusted to 20 μM. The binding reaction between fusion protein to ABA was detected by ITC. To ensure the univariate of the experiment, an ITC buffer was used to prepare an ABA solution with 20 times protein concentration to ensure the matching of buffer environment between the two. The ITC analysis used 20-μM fusion protein and 400-μM ABA in an ITC200 calorimeter (MicroCal). Furthermore, to determine the binding strength of FaRRP1 with FaPYL2 and FaABAR, the final concentration of the FaRRP1 fusion protein was adjusted to both 20 μM and 200 μM, FaPYL2 fusion protein was adjusted to 200 μM, and FaABAR fusion protein was adjusted to 20 μM. Subsequently, 20 μM FaRRP1 in 200 μM FaPYL2 drops as a FaRRP1 binding to FaPYL2 assay and 20 μM FaABAR in 200 μM FaRRP1 drops as a FaRRP1 binding to FaABAR assay. At the same time, 20 μM FaRRP1 200 μl was gently mixed with 20 μM FaPYL2/FaABAR 200 μl at 4 °C for 30 min and titrated with 400 μM ABA. The reaction parameters were as follows: cell temperature, 25 °C; reference power, 10 μ cal/sec; initial delay, 60 s; agitator speed, 1000 rpm/min; and titration times, 20 times. The volume of the first drop was 0.5 μl, and then the volume of each slide was 2 μl, with a total of 20 drops. The experiment was repeated three times. Data fitting was performed using ORIGIN 7.0 software (MicroCal) (https://www.originlab.com/).

### Y2H assays

The coding sequence (CDS) of FaRRP1 was cloned into the pGADT7 vector. The CDSs of FaABAR and FaPYL2 were cloned into the pGBKT7 vector. The Y2HGold Chemically Competent Cell for the yeast transformation Kit following the manufacturer's instructions (WEIDI) for experimental manipulation. Selected on synthetic medium (SD) SD-Trp/Leu and SD-Trp/Leu/His/Ade. The primer sequences used are listed in Table S1.

### BiFC assays

The construction of pSPYNE-FaPYL2, pSPYNE-FaABAR, and pSPYCE-FaRRP1 recombinant plasmids was transformed into *Agrobacterium* GV3101 (WEIDI). *Agrobacterium* cultures were resuspended in infiltration buffer (100 mM MES [pH 5.6], 100 mM MgCl2, and 200 mM acetosyringone) to a final A_600_ of 0.6. pSPYNE-FaPYL2 and pSPYCE-FaRRP1, pSPYNE-FaABAR, and pSPYCE-FaRRP1 in equal proportions were transformed into 4- to 6-week-old *N. benthamiana* leaves. Three days later, the lower epidermal cells of the leaves were observed under the laser confocal microscope. The experiment was repeated three times. The primer sequences used are listed in Table S1.

### GST pull-down assays

Protein with a His-Tag and GST-Tag was expressed and purified for GST pull-down assay. FaPYL2-His and FaABAR-His protein was added to GST-FaRRP1 or GST protein and incubated at 4 °C for 2 h, followed by washing four or five times with pull-down buffer (20 mM Tris–Hcl, 150 mM NaCl, 1 mM β-mercaptoethanol, 3 mM EDTA [pH 8.0], 1% NP-40). The proteins bound to the particles were eluted and separated with SDS-PAGE and visualized using a Western blotting assay. The primer sequences used are listed in Table S1.

### Construction of the RNAi and overexpression plasmids used in transgenic fruit

To construct the FaRRP1-RNAi, FaRRP1-OE, FaPYL2-RNAi, FaPYL2-OE, FaABAR-RNAi, and FaABAR-OE vectors, Gateway BP ClonaseTMⅡEnzyme Mix Kit and Gateway LR ClonaseTMⅡEnzyme Mix Kit (Invitrogen) was used. The constructed RNAi and OE vectors were transferred into GV3101 competent cells (WEIDI). Based on the *Agrobacterium*-mediated method, a syringe was used to inject the isolated strawberry fruit at the DG stage with *Agrobacterium tumefaciens*. The primer sequences used are listed in Table S1.

### *In vitro* incubation of fruit discs

*In vitro* treatments were performed on fruits in the white stage using 100 μM ABA, and ABA-free buffer incubation was used as the control. The incubation was done as described by Song *et al*. (77). After a 2-h incubation followed by washing with double-distilled water, the discs were frozen in liquid nitrogen and kept at −80 °C until use. Samples are collected once at 0 h, 1 h, and 2 h. The experiment was performed with three replicates.

### RT-qPCR analysis

Total RNA was isolated from the fruit using the aforementioned method. The first-strand cDNA was used as the template for RT-qPCR, which was performed using the Trans Start Top UreenqPCR Super Mix Kit (TransGen Biotech) on the Light Cycler 96 real-time PCR system, with cDNA under different treatments as the template and strawberry *Actin* as the internal reference gene. The reaction procedures were as follows: predenaturation at 94 °C for 30 s, followed by 40 cycles of denaturation at 94 °C for 5 s, annealing at 60 °C for 15 s, and extension at 72 °C for 10 s. The experiment was duplicated three times independently. The *Actin* of the standard sample and the cDNA of the sample to be tested were repeated three times. Relative expression values were calculated by the 2^−ΔΔCt^ method, and SPSS22.0 software (https://www.spss.com) was used to analyze the experimental data. The primer sequences used are listed in Table S1.

### Subcellular localization analysis

The CDSs of FaRRP1, FaPYL2, and FaABAR were amplified and inserted into the Super1300-GFP vector, respectively. The recombinant vector was transferred into GV3101 (WEIDI). *Agrobacterium* cultures were resuspended in infiltration buffer (100 mM MES [pH 5.6], 100 mM MgCl2, and 200 mM acetosyringone) to a final A_600_ at 0.6. The vectors were transformed into 4- to 6-week-old *N. benthamiana* leaves. Among them, Super1,300-GFP and 1300-Mcherry were the control group; cytoplasmic-marker and FaPYL2-GFP mixed samples were injected into lower epidermal cells of *N. benthamiana* leaves, also mixing chloroplast-marker and FaABAR-GFP samples. Three days later, the more deficient epidermal cells of *N. benthamiana* leaves were observed under the laser confocal microscope. In addition, 10 μM DAP1 working solution was prepared with PBS Buffer. FaABAR-GFP–infected *N. benthamiana* leaves were put into the working solution, stained for 20 min, and washed 3 times with PBS Buffer. The lower epidermal cells of *N. benthamiana* leaves were observed. The experiment was repeated three times. The primer sequences used are listed in Table S1.

### Endocytosis experiments under ABA treatment

The 10-days-old *FaRRP1* OE and WT Arabidopsis seedlings growing under the normal condition were collected and soaked into the equilibrium solution [1/2 MS salt (pH=5.70), 1% sugar (w/v)] for 30 min. Then, the seedlings were treated with 0.1 μM ABA. After 48 h later, these seedlings were stained with 5 μM FM4-64 (Invitrogen) dye for 10 min, washed with equilibrium solution for 3 times, and root cells were observed under a confocal laser scanning microscope (Zeiss LSM 710 META). The experiment was repeated three times.

### Statistical analysis

All data are presented as means of at least three independent biological replicates ± SD. The data were analyzed with one-way ANOVA followed by Duncan’s multiple comparison tests or Student’s *t* test. *p* Values less than 0.05 are considered significant and plotted. For all graphs ∗*p* ≤ 0.05, ∗∗*p* ≤ 0.01. Graphs were edited in Photoshop CS (Adobe).

## Data availability

The sequences reported in this article are available in the GenBank database with accession numbers JQ619656.1, LOC101306195, and LOC101311885. CAS number for ABA: 14375-45-2. Other data presented in this article are stored with us and available for sharing upon request.

## Supporting information

This article contains supporting information.

## Conflict of interest

The authors declare that they have no conflicts of interest with the contents of this article.
