# Supplementary material for: A clathrin-related protein FaRRP1/SCD2 integrates ABA trafficking and signaling to regulate strawberry fruit ripening
Source: J Biol Chem. 2023 Sep 13;299(10):105250. doi: 10.1016/j.jbc.2023.105250 (PMC10582773; doi:10.1016/j.jbc.2023.105250)
Supplement: Supplemental Table S1 [file mmc1.docx]

**Supplemental Table 1 Primers used in this study**

| Primer name | Sequences for PCR |
| --- | --- |
| *FaPYL2*-F  *FaPYL2*-R | 5′- ATGTTTCCGATGGAGTCGAGC -3′  5′- TTCATGTCCACCCCCATGCAG -3′  5′-GGGGACAAGTTTGTACAAAAAAGCAGGCTTCAGGAGCTTTCCGATCTTGAGC-3′  5′-GGGGACCACTTTGTACAAGAAAGCTGGGTCTTACCTCCCTTATGCTGCCGA-3′  5′-GGTCGCGGATCCGAATTCATGTTTCCGATGGAGTCGAGC -3  5′- GGCCGCAAGCTTGTCGACTTCATGTCCACCCCCATGCAG -3′ |
| RNAi*-FaPYL2*-F  RNAi*-FaPYL2*-R |  |
| pET28a*-FaPYL2*-F  pET28a*-FaPYL2*-R |  |
| Qt*-FaPYL2*-F  Qt*-FaPYL2*-R | 5′- ACAAATTCGAACCCTCCCCC -3′  5′- AGACGACGGTTACCTCCCTT -3′  5′- CAGGGGCCCGGGGTCGACATGTTTCCGATGGAGTCGAGC -3′  5′- GGTACCGGATCCACTAGTTTCATGTCCACCCCCATGCAG -3′ |
| Super1300*-FaPYL2*-F  Super1300*-FaPYL2*-R |  |
| *Actin*-F | 5′ -TGCATATATCAAGCAACTTTACACTGA-3′ |
| *Actin*-R | 5′ -ATAGCTGAGATGGATCTTCCTGT-3′ |
| OE*-FaPYL2*-F  OE*-FaPYL2*-R | 5′-GGGGACAAGTTTGTACAAAAAAGCAGGCTTCAGGAGCTTTCCGATCTTGAGC-3′  5′-GGGGACCACTTTGTACAAGAAAGCTGGGTCTTACCTCCCTTATGCTGCCGA-3′ |
| pSPYNE*-FaPYL2*-F  pSPYNE*-FaPYL2*-R | 5′-TGGCGCGCCACTAGTGGATCCATGTTTCCGATGGAGTCGAGC -3′  5′-GTCGACCTCGAGGGTACCGCTTTCATGTCCACCCCCATGCAG -3′ |
| BD-*FaPYL2*-F  BD*-FaPYL2*-R | 5′- TCAGAGGAGGACCTGCATATGATGTTTCCGATGGAGTCGAGC -3′  5′- TCGACGGATCCCCGGGAATTCTTCATGTCCACCCCCATGCAG -3′ |
| *FaRRP1*-F  *FaRRP1*-R | 5'- ATGGATCGGAGGAGACAAGCG -3'  5'-TAATGAAGAGGTGTCGAAATCTGCGG-3' |
| RNAi*-FaRRP1*-F  RNAi*-FaRRP1*-R | 5'-GGGGACAAGTTTGTACAAAAAAGCAGGCTTCTAGCTGTGCAGCATGCCATA -3'  5'-GGGGACCACTTTGTACAAGAAAGCTGGGTCTCCCCAGTGAGGTCACTCAA-3' |
| pMAL-c5x-His*-FaRRP1*-F  pMAL-c5x-His-*FaRRP1*-R | 5’-GACTAATTCGAGCTCATGGATCGGAGGAGACAAGCGA-3’  5’-ATTTAGAATTCATGATGATGATGATGGTGTAATGAAGAGGT-3’ |
| Qt*-FaRRP1*-F  Qt*-FaRRP1*-R | 5'- ATGAGGACGGGAAAGATA -3'  5'- GCTAAAGATGACCAATGT -3' |
| Super1300*-FaRRP1*-F  Super1300*-FaRRP1*-R | 5'-CTGCAGGGGCCCGGGGTCGACATGAGGACGGGAAAGATA-3'  5'-CATGGTACCGGATCCACTAGTTAATGAAGAGGTGTCGAAATCTGCGG-3' |
| OE*-FaRRP1*-F  OE*-FaRRP1*-R | 5'-GGGGACAAGTTTGTACAAAAAAGCAGGCTTCTAGCTGTGCAGCATGCCATA -3'  5'-GGGGACCACTTTGTACAAGAAAGCTGGGTCTCCCCAGTGAGGTCACTCAA-3' |
| pSPYCE*-FaRRP1*-F  pSPYCE*-FaRRP1*-R | 5'-TGGCGCGCCACTAGTGGATCCATGGATCGGAGGAGACAAGCG -3'  5'-GTCGACCTCGAGGGTACCGCTTAATGAAGAGGTGTCGAAATCTGCGG-3' |
| AD-*FaRRP1*-F  AD*-FaRRP1*-R | 5'- GCCATGGAGGCCAGTGAATTCATGGATCGGAGGAGACAAGCG -3'  5'- CAGCTCGAGCTCGATGGATCCTAATGAAGAGGTGTCGAAATCTGCGG-3' |
| pGEX4T-1*-FaRRP1*-F  pGEX4T-1*-FaRRP1*-R | 5'-CCGCGTGGATCCCCGGAATTCATGGATCGGAGGAGACAAGCG -3'  5'-GATGCGGCCGCTCGAGTCGACTAATGAAGAGGTGTCGAAATCTGCGG-3' |
| *FaABAR*-F  *FaABAR*-R | 5'-ATGGCTTCTCTAGTCTCCTCAC-3'  5'-TCGATCAATTCCCTCGATTTTGTC-3' |
| RNAi*-FaABAR*-F  RNAi*-FaABAR*-R | 5'-GGGGACAAGTTTGTACAAAAAAGCAGGCTTCGCAACCCGGATCTTCTCCAA-3'  5'-GGGGACCACTTTGTACAAGAAAGCTGGGTCCATCAGCTGTGCTCAATGCC-3' |
| pET28a*-FaABAR1*-F  pET28a*-FaABAR*-R | 5'-CGAATTCGAGCTCCGTCGACATGGCTTCTCTAGTCTCCTCAC-3'  5'-TGGTGGTGGTGGTGCTCGAGTCGATCAATTCCCTCGATTTTGT-3' |
| Qt*-FaABAR*-F  Qt*-FaABAR*-R | 5'- TCCTCATGGAATGATGAGAAGC -3'  5'- GTGGTGGTGTCAGCAATGTAAG -3' |
| Super1300*-FaABAR*-F  Super1300*-FaABAR*-R | 5'-CTGCAGGGGCCCGGGGTCGACATGGCTTCTCTAGTCTCCTCAC-3'  5'-CATGGTACCGGATCCACTAGTTCGATCAATTCCCTCGATTTTGTC-3' |
| OE*-FaABAR*-F  OE*-FaABAR*-R | 5'-GGGGACAAGTTTGTACAAAAAAGCAGGCTTCGCAACCCGGATCTTCTCCAA-3'  5'-GGGGACCACTTTGTACAAGAAAGCTGGGTCCATCAGCTGTGCTCAATGCC-3' |
| pSPYNE*-FaABAR*-F  pSPYNE*-FaABAR*-R | 5'-TGGCGCGCCACTAGTGGATCCATGGCTTCTCTAGTCTCCTCAC-3'  5'-GTCGACCTCGAGGGTACCGCTTCGATCAATTCCCTCGATTTTGTC-3' |
| BD-*FaABAR*-F  BD*-FaABAR*-R | 5'-TCAGAGGAGGACCTGCATATGATGGCTTCTCTAGTCTCCTCAC-3'  5'-TCGACGGATCCCCGGGAATTCTCGATCAATTCCCTCGATTTTGTC-3' |
| Qt-*FaAP1*-F  Qt-*FaAP1*-R | 5'-TGAGTACGCAGGTCAAACCG-3'  5'-AGTGCGTCATTTTCAGGTGC-3' |
| Qt-*FaAP2*-F  Qt-*FaAP2*-R | 5'-TCCATCCCCGACTCAAATGC-3'  5'-CACTCATCGACAGCGTGAGA-3' |
| Qt-*FaCLC1*-F  Qt-*FaCLC1*-R | 5'-CTCCACCACACATGATGCCT-3'  5'-AGCCTGCTCAACTGTTGGTT-3' |
| Qt-*FaCLC2*-F  Qt-*FaCLC2*-R | 5'-TACTGGAAGGCAGTTGCAGA-3'  5'-GTGGCGAAGGCTTTAGGTGA-3' |
| Qt-*FaSCD1*-F  Qt-*FaSCD1*-R | 5'-TGATGCTAACGATGCGACGA-3'  5'-GGTGACCGCGAAACTAGACA-3' |
| Qt-*FaSCD2*-F  Qt-*FaSCD2*-R | 5'-AGCCACATAAGCCGTAGCAG-3'  5'-AGAGGTGTCGAAATCTGCGG-3' |
| Qt-*FaCHC1*-F  Qt-*FaCHC1*-R | 5'-GGAAATGAGGCGTGTTGCTG-3'  5'-AGACAAAGAGGCATGACGCA-3' |
| Qt-*FaCHC2*-F  Qt-*FaCHC2*-R | 5'-CCTTCGCCCAGTACAAGGTT-3'  5'-ATCTGCATGGCAACTGGGAA-3' |
